# US postmarketed drug removals: Analysis of discontinued and withdrawn drugs

**DOI:** 10.1016/j.jpet.2026.104996

**Published:** 2026-07-31

**Authors:** Lilly Josephine Bindel, Roland Seifert

**Affiliations:** Institute of Pharmacology, Hannover Medical School, Hannover, Germany

**Keywords:** Drug safety, Pharmacovigilance, US Food and Drug Administration, United States, Withdrawal, Boxed warnings

## Abstract

Drugs removed from the US market after approval can be either discontinued by the manufacturer or withdrawn by the US Food and Drug Administration (FDA). However, a systematic overview is currently lacking. This minireview provides a comprehensive analysis of removed drugs, identifies patterns regarding drug classes, indications, adverse effects, and scientific interest, and compares US removals with the European Union and FDA boxed warnings. A total of 166 drugs were removed between 1955 and 2026, including 88 discontinued and 78 withdrawn drugs. Removed drugs represented a wide range of therapeutic fields, with certain drug classes occurring repeatedly, particularly cyclooxygenase inhibitors and fluoroquinolones. Market removals were frequently associated with serious adverse effects such as hepatotoxicity, cardiotoxicity, and hematologic toxicity. The prominent drug classes and adverse effect categories also occurred frequently among drugs carrying boxed warnings. While withdrawn drugs were typically banned by the FDA because of severe safety concerns, discontinued drugs were often removed because of commercial decisions or obsolescence, sometimes in combination with safety considerations. The analysis of publication peaks showed that for most drugs, scientific interest peaked before market removal, while in other cases, publication peaks occurred around or after withdrawal, indicating renewed scientific interest. Drug removals from the US market represent a heterogeneous group of drugs, indications, and underlying causes. While withdrawals and discontinuations differ in their regulatory implications, they share overlapping characteristics and safety considerations. Integration of European Union availability and FDA boxed warning comparisons highlights international differences and patterns requiring precautionary attention, with recurring drug classes and adverse effects.

**Significance Statement:**

This minireview provides a comprehensive overview of US postmarketing drug removals, covering drugs and causes, as well as evaluating and comparing their characteristics. Patterns and characteristics are identified and compared between discontinued and withdrawn drugs. The analysis of publication peaks and the derived patterns allow an evaluation of changes in scientific interest. This analysis provides information on past drug safety decisions and guides future risk-benefit assessments, while identifying prevalent drug classes and adverse effects that require precaution.

## 1 Introduction

Drugs that are removed from the US market after US Food and Drug Administration (FDA) approval can be either discontinued by their manufacturer or withdrawn by the FDA. Reasons are primarily related to safety or efficacy issues that are sufficiently severe to prevent continued pharmacotherapeutic use, as well as commercial or business-related discontinuations.^1^, ^2^, ^3^ Regulatory agencies provide multiple resources on drug safety, including FDA drug safety communications, the Orange Book, and online data bases such as Inxight Drugs.^4^ However, a comprehensive and up-to-date overview of withdrawn or discontinued drugs for the US market is currently missing. Bioinformatic projects such as Withdrawn 2.0^5^ are available but remain incomplete and fragmented. Moreover, most literature addresses specific therapeutic fields, limited timeframes, or non-US markets.^6^, ^7^, ^8^

This minireview aims to provide a systematic and comprehensive overview of drugs removed from the US market, including both FDA-withdrawn drugs and manufacturer-discontinued drugs. The analysis included prescription drugs that had previously received FDA approval for human use. Excluded were drugs that were never approved by the FDA for human use, substances without a therapeutic effect (eg, excipients), or drugs that were not fully withdrawn (eg, because application forms, dosages, or indications remained available). For the initial collection of drugs, the following sources were consulted: Withdrawn 2.0 (last accessed: March 16, 2026),^5^ §216.24 “Drug products withdrawn or removed from the market for reasons of safety or effectiveness” of the Federal Food, Drug, and Cosmetic Act (last accessed: March 17, 2026),^9^ and FDA Drug Safety Communications for the years 2023–2026 (last accessed: March 17, 2026).^10^ To account for the limited recency and completeness of these sources, the approval status of all collected drugs was verified using the FDA Orange Book (last updated: December 31, 2025).^2^ Figure 1 provides an overview of the methodological approach.

**Fig. 1:**
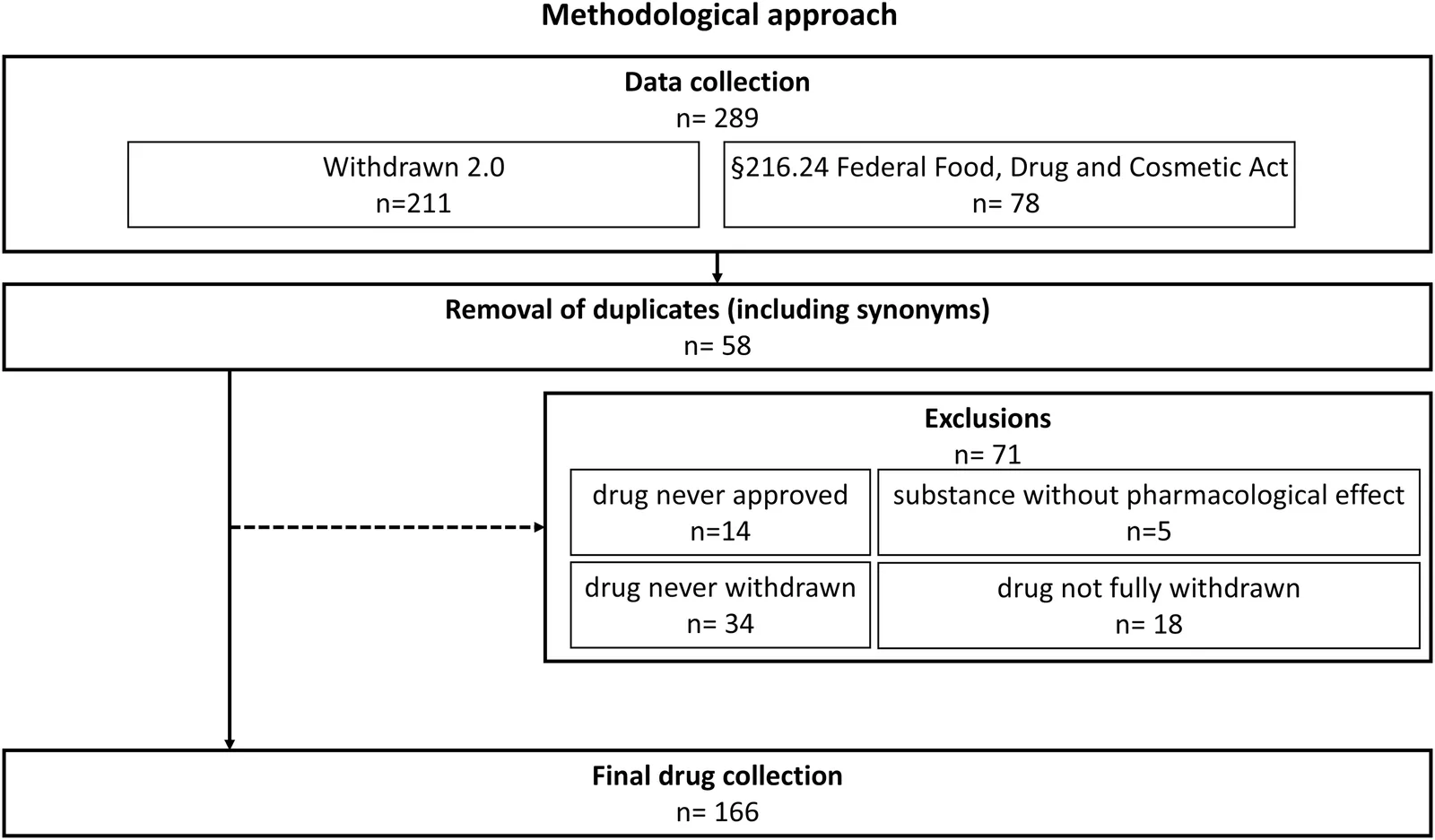
Methodological approach of the data collection for drugs withdrawn or discontinued from the US market.

By analyzing characteristics, patterns, and publication trends, this analysis highlights frequently affected drug classes, common adverse effects, and differences between withdrawn and discontinued drugs. In addition, US drug removals are compared with the European Union (EU) market to evaluate country-specific differences. Furthermore, the characteristics of removed drugs are compared with FDA boxed warnings^11^ to identify drugs and drug classes for which precautionary attention is particularly warranted, thereby revealing patterns relevant to risk assessment and future drug safety considerations.

## 2 Historical background, knowledge gaps, and recent advances

Analyses of drug removals are limited. Available studies have often focused on single therapeutic areas, restricted timeframes, or have not been specific to the US market, providing only partial insight into postmarket safety and regulatory outcomes.^1^^,^^6^ Bioinformatic resources such as Withdrawn 2.0^5^ provide preliminary datasets but remain fragmented and incomplete. These limitations hindered the identification of recurring characteristics and patterns in the risk-benefit assessment of drugs.

Previous work from our group investigated drug approvals and their postmarketing development, examined prescription trends of widely used and obsolete medicines, the changing pharmacological importance of individual drugs, and the evaluation of therapeutic drug classes such as monoclonal antibodies or protein kinase inhibitors, and country-specific differences in regulatory decisions and prescribing practices.^12^, ^13^, ^14^, ^15^, ^16^, ^17^, ^18^, ^19^ More recent analyses focused on FDA boxed warnings, providing a structured categorization of adverse effects and drug classes and identifying prevalent therapeutic areas and drug classes.^11^ These studies demonstrate how systematic analyses can reveal insights and patterns not apparent from individual drug evaluations.

Knowledge of drug removals and the underlying reasons is important for understanding safety issues that are of particular relevance. By examining persistent drug characteristics, key adverse effects, and aspects critical to the assessment of a drug’s risk-benefit ratio, this information can guide future considerations in drug safety. Moreover, the availability of a comprehensive overview of withdrawn and discontinued drugs enables international comparisons, highlights country-specific regulatory differences, and supports the standardization and harmonization of regulatory practices across markets and countries.

## 3 Time course of US market drug removals

From 1955 to 2026, 88 drugs were discontinued and 78 were withdrawn, resulting in a total of 166 drugs that were removed from the US market. Although there is no dominant trend across the entire period, removals peaked during the 2000s. For 4 drugs, no exact year of market removal was found.

In the early time period, from 1961 to 1978, withdrawals dominated. Conversely, in the later time period, from 1994 to 2021, discontinuations dominated. While in some years only discontinuations (eg, 1955, 1990, and 2013) or withdrawals (eg, 1961, 1970, and 1978) were observed, both types of removal were reported in many years (eg, 1963, 1983, 2001, and 2011).

The highest annual numbers of removed drugs occurred in 2001 and 2006, with 8 drugs removed in each year. For discontinued drugs, the largest number, 7, was removed in 2006. For withdrawn drugs, the highest number, 5, occurred in both 1970 and 2000. A graphical visualization is provided in Fig. 2.

**Fig. 2:**
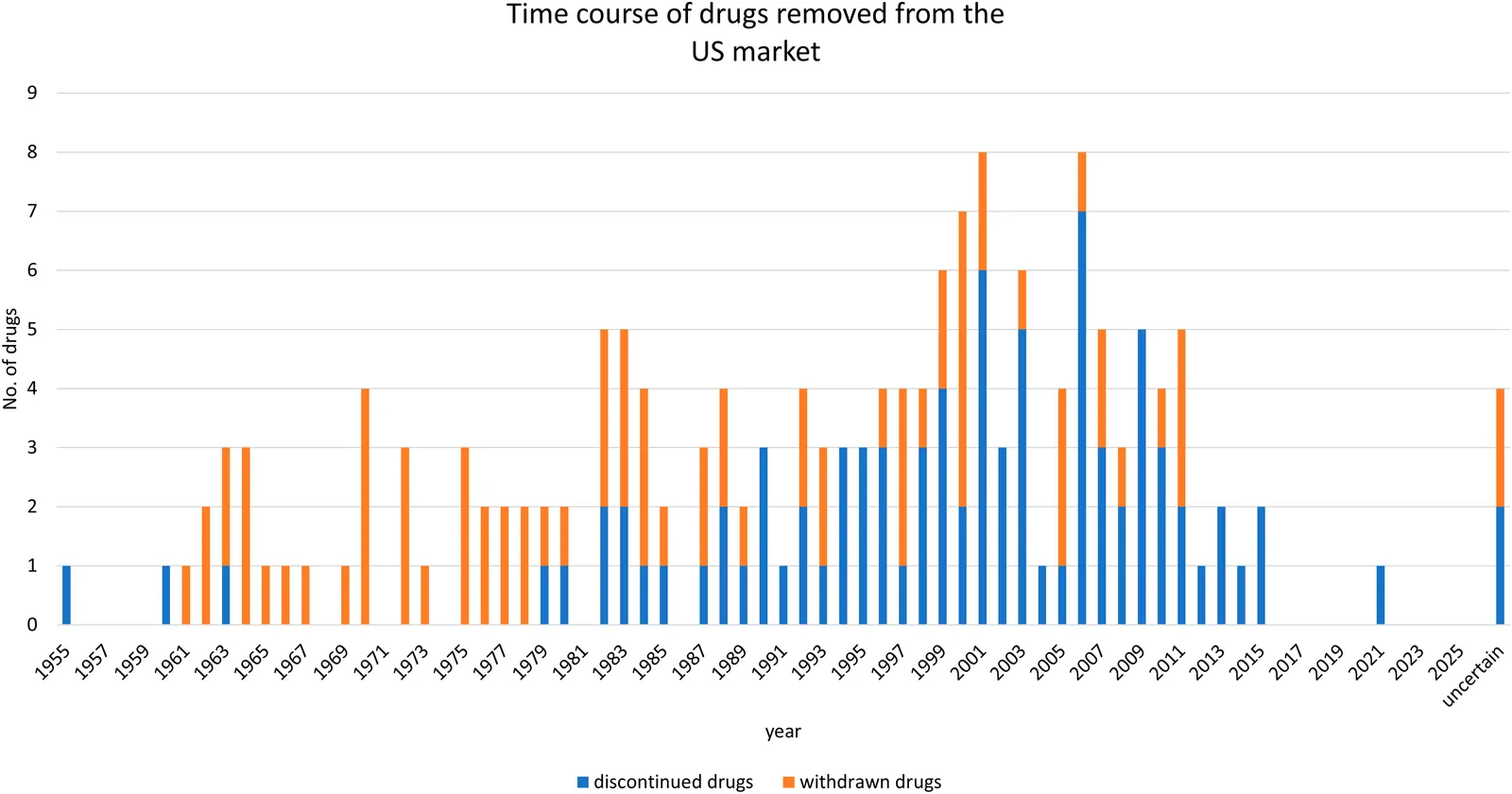
Time course of the number of drugs removed from the US market. On the *x*-axis, the respective years is presented, while the *y*-axis represents the number of drugs that were removed. The last bar on the right gives the number of drugs where the exact removal year is uncertain. Withdrawn drugs are highlighted in orange, and discontinued drugs are highlighted in blue.

## 4 Differentiation between drug withdrawals versus discontinuations and their reasons

When analyzing drugs that were removed from the US market, it is important to differentiate between their legal status, because this has implications for the reasons for removal and the potential reinstatement of a drug.^2^^,^^20^^,^^21^ Table 1 provides an overview of characteristics and differentiates between drug discontinuations and withdrawals.

**Table 1:** Key characteristics of discontinued and withdrawn drugs that were removed from the US market

Characteristic	Discontinued Drugs^1^, ^2^, ^3^^,^^20^	Withdrawn Drugs^1^^,^^2^^,^^9^^,^^10^
Responsible authority	Manufacturer	FDA
Regulatory action	Marketing voluntarily stopped by the manufacturer	Drug banned from the market by the FDA
Approval status after drug removal	Approval generally remains valid after investigation of FDA whether not discontinued because of safety/efficacy reasons (otherwise withdrawal by the FDA likely)	FDA approval withdrawn
Listing in FDA Orange Book	Listed in the “Discontinued Drug Product List”	Not listed
Possibility of market return	Relatively easy reintroduction if marketing is resumed	Requires resubmission and regulatory review
Main reasons for removal	Often commercial or business decisions; may also include safety or efficacy considerations	Severe safety or efficacy issues
Typical context	Multifactorial evaluation of risk-benefit ratio and declining clinical use	Strong safety signal leading to regulatory intervention
Common removal reasons	Often multiple contributing factors	Frequently one dominant safety concern
Drug examples	Methicillin, quinethazone, tridihexethyl	Aminophenazone, butamben, trovafloxacin

Given are important aspects that explain and differentiate between the 2 types of drug removal. A comprehensive and detailed overview about drug characteristics and removal reasons can be found in Supplemental Tables 1 and 2.

Discontinued drugs are no longer marketed by their manufacturer. Reasons include business decisions and commercial considerations, meaning that marketing is no longer financially viable. This can be due to declining prescriptions because of the availability of more favorable options, as in dydrogesterone or mazindol,^22^^,^^23^ or patent expiration, as seen for metipranolol.^4^ Changes in therapeutic relevance over time, including transitions from widely used to obsolete drugs, have also been described in further analyses of prescription trends and historical drug evaluations.^12^^,^^14^^,^^15^ Another reason may be a political decision, for example, in the case of thiopental, which was removed by the manufacturer because of its use in the execution of death sentences in US prisoners.^24^ Safety issues are another important reason for discontinuation. In such cases, the manufacturer voluntarily removes the drug from the market, as observed for pirprofen, alatrofloxacin, and sitaxentan because of hepatotoxicity.^1^^,^^4^^,^^24^^,^^25^ Furthermore, manufacturing issues, for example, with aminopterin,^4^^,^^26^ can also result in a manufacturer’s decision to discontinue a drug. These findings indicate that pharmacological vulnerability, the extent and context of market use, and the available regulatory pathway can lead from a pharmacological disadvantage to commercial discontinuation. Sometimes, it is not possible to determine the exact reason because a drug may be removed as a business decision in which adverse effects contribute to the drug’s inferiority but may not be the main reason.^1^ Often, no exact reason was stated by the manufacturer, as for iopanoic acid.^4^^,^^25^

Withdrawn drugs are banned from the market by the FDA. The main reason for this action is a severe safety issue, as in chlornaphazine, chloroform, danthron, oxeladin, and phenolphthalein with a carcinogenic risk.^3^^,^^4^^,^^25^^,^^27^^,^^28^ Here, the manufacturer has no choice but to stop marketing the drug. Here, the regulatory consequence is dominated by a formal assessment that continued use is no longer acceptable. Many of these drugs are listed in the 2018 version of the “Federal Food, Drug, and Cosmetic Act” (§216.24).^9^

This distinction has important legal consequences. A drug that was discontinued for reasons other than safety or efficacy remains approved and is listed in the “discontinued drug product list” of the FDA Orange Book,^2^ making a return to marketing relatively easy. In contrast, a withdrawn drug has had its approval withdrawn, and the drug cannot be marketed in the US again without a major resubmission and review. Withdrawn drugs are not listed in the FDA Orange Book.^2^ The FDA often evaluates whether a drug was discontinued because of safety or efficacy reasons or because of a business decision.^29^, ^30^, ^31^, ^32^, ^33^, ^34^, ^35^, ^36^, ^37^ This evaluation may result in a withdrawal of the drug’s approval, after which it is no longer listed in the FDA Orange Book.

In this analysis, classification is based on who initially removed a drug. Supplemental Tables 1 and 2 provide an overview of all removed drugs and the associated reasons. The status is given as discontinued if marketing was stopped by the manufacturer and as withdrawn if the drug was banned by the FDA. Furthermore, the reason why a drug was discontinued was evaluated (business decision, safety, and efficacy).

## 5 Characterization of removed drugs

Removed drugs were broadly allocated to different usage fields (analgesics and immunomodulators; anti-infective drugs; cardiovascular and renal drugs; central nervous system (CNS); cytotoxic treatment; diagnostic drugs; endocrine system; and metabolism, gastrointestinal, and respiratory system), and for each, the FDA-approved indication is provided. In the following, the drugs are analyzed according to their usage field, drug class, and indication, while a comprehensive overview is provided in Supplemental Table 3. Figure 3 provides an overview of characteristics of removed drugs (eg, mechanism of action, usage field, and removal reason), while Fig. 4 visualizes the number and distribution of prevalent drug classes among US market removals.

**Fig. 3:**
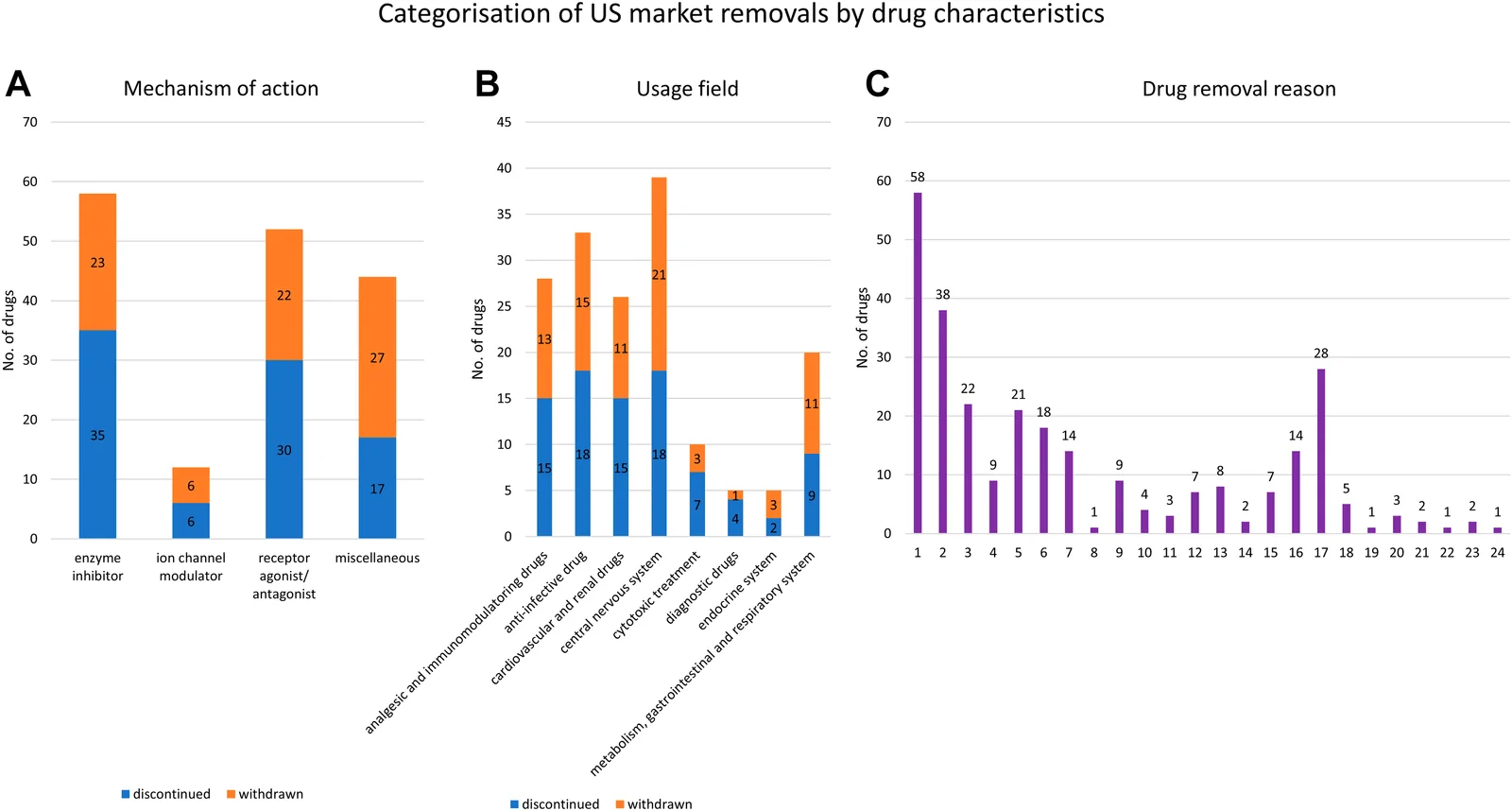
Overview of characteristics of removed drug classes. Each drug was categorized by (a) its mechanism of action, (b) its usage field, and (c) its drug removal category. A differentiation is provided between the number of discontinued (blue-colored) and withdrawn (orange-colored drugs). While each drug was assorted to one category for its mechanism (a) and usage field (b), one drug may be listed for multiple drug removal reasons (c). The following market removal reason categories were assigned: (1) business decision, commercial reasons; (2) availability of better treatment options (more efficient or safe), obsoleteness; (3) hepatotoxicity and hepatic adverse effects; (4) nephrotoxicity and renal adverse effects; (5) hematologic toxicity and hematologic adverse reactions; (6) immunologic and hypersensitivity reactions; (7) dermatologic toxicity and cutaneous adverse reactions; (8) ototoxicity; (9) carcinogenicity and induction of malignancies; (10) drug or food interactions; (11) metabolic and endocrine adverse effects, and laboratory abnormalities; (12) tolerance, dependence, misuse, and withdrawal symptoms; (13) gastrointestinal toxicity and gastrointestinal adverse effects; (14) ophthalmic toxicity and visual disturbances; (15) psychiatric and behavioral adverse effects; (16) neurotoxicity and neurological adverse effects; (17) cardiotoxicity and cardiac adverse effects; (18) pulmonary toxicity and respiratory adverse effects; (19) developmental, reproductive, and embryo-fetal toxicity; (20) increased mortality or fatal outcomes; (21) exacerbation or worsening of underlying disease; (22) political decisions of the manufacturer; (23) musculoskeletal toxicity; and (24) production difficulties by the manufacturer.

**Fig. 4:**
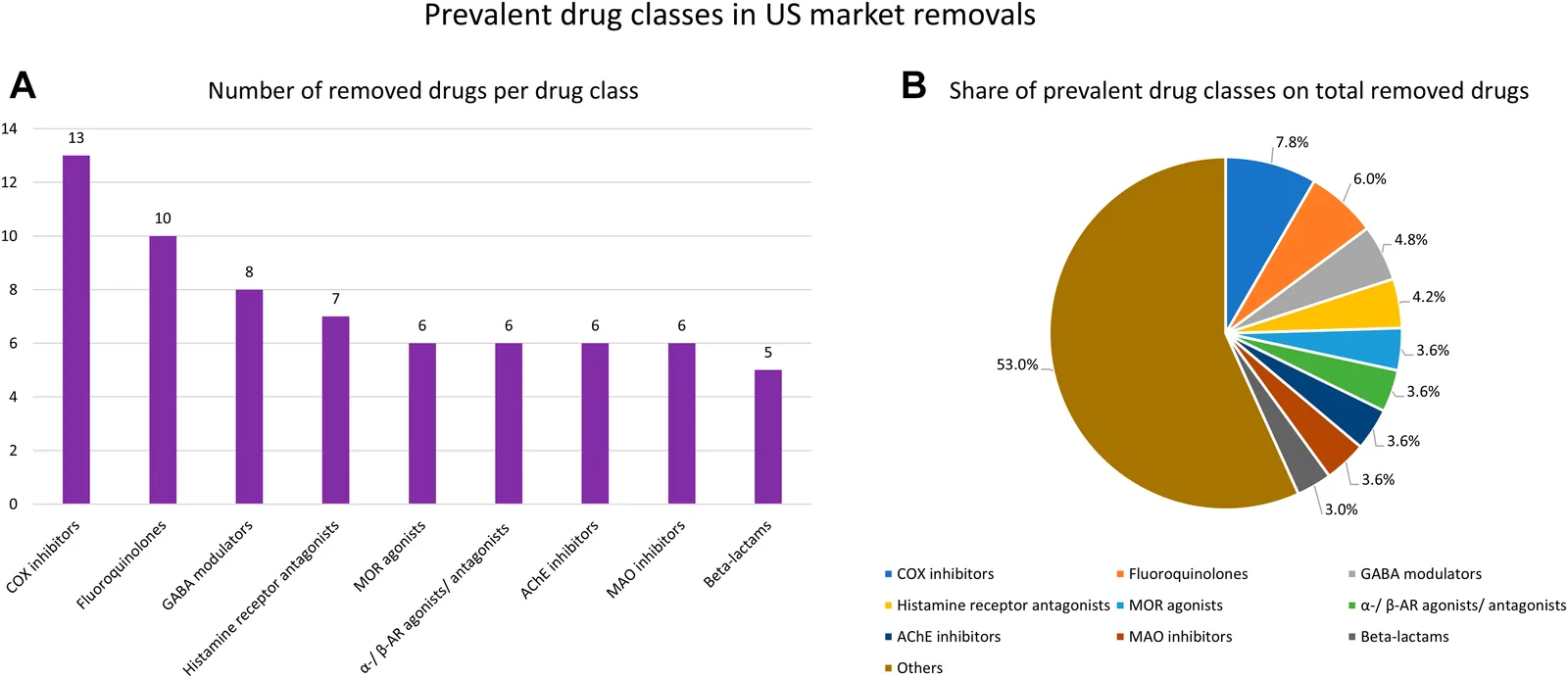
Overview of prevalent drug classes, being listed with 5 or more drugs in removed drug classes. Provides is in (a) their absolute number per drug class, and in (b) their share on the total number of US market removals. Drug classes are sorted by their largest number of listings.

For analgesic and immunomodulating drugs, 28 drugs are listed. The most prevalent indications were pain, rheumatic disorders, and allergic symptoms. Prevalent drug classes are cyclooxygenase (COX) inhibitors (*n* = 13), histamine receptor 1 antagonists (*n* = 7), µ-opioid receptor agonists (*n* = 6), and pyrazolones (*n* = 3). Reasons for market removal are diverse, ranging from cardiovascular adverse effects (heart attack, stroke, and arrhythmia) to hematologic adverse events (hemolytic/aplastic anemia, neutropenia, agranulocytosis, and bleeding) to organ toxicities (hepatotoxicity and nephrotoxicity), and hypersensitivity reactions.

The anti-infective group comprises 33 drugs, many of them used for bacterial, viral, or further infections. Frequently listed drug classes include fluoroquinolones (*n* = 10) and *β*-lactams (*n* = 5), among others. Reasons for market removal include organ toxicities (hepatotoxicity, ototoxicity, cardiac toxicity, hematologic toxicity, photosensitivity, and neurotoxicity) and hypersensitivity reactions (including Stevens–Johnson syndrome).

The cardiovascular and renal group includes 26 drugs, most of them used for hypertension, thromboembolic disorders, and cardiac arrhythmia. Frequent drug classes include *α*-/*β*-adrenoceptor agonists/antagonists (*n* = 6), sodium channel blockers (*n* = 4), calcium channel blockers (*n* = 3), and sodium-chloride cotransporter/ sodium-potassium-chloride cotransporter inhibitors (*n* = 3). Prevalent adverse effects include cardiac (arrhythmia, myocardial infarction, and hypertension) and hematologic (stroke, bone marrow suppression, and bleeding) events, as well as increased mortality.

The CNS comprises 38 drugs, often used in anesthesia, epilepsy, and depression. Many removed drugs belong to GABA receptor antagonists (*n* = 8) and achetylcholinesterase inhibitors (*n* = 6), as well as monoamine oxidase inhibitors (*n* = 5). Adverse effects range from cardiac issues (arrhythmia, cardiac fibrosis, and heart attack) to hematologic (agranulocytosis and aplastic anemia), hepatic, neurological (including Guillain–Barré syndrome), neuropsychiatric (suicidal ideation, hallucinations, dependence, and withdrawal symptoms), and hypersensitivity reactions.

In cytotoxic treatment, 10 drugs are listed. They were frequently used for skin disorders (psoriasis and vitiligo) and hematologic cancers (leukemia and lymphoma). The listed drug classes are diverse, with DNA alkylating agents listed twice. Adverse effects include a risk of induced malignancy, organ toxicities (cardiac, hepatic, renal, and CNS), and hematologic adverse events (thrombosis, agranulocytosis, and granulocytopenia).

The diagnostic-drug and the endocrine-system group consist of 5 drugs, mainly used for diagnostic procedures, hormone substitution, and contraception. Once again, the included drug classes are diverse. Reasons for market removal among diagnostic drugs include gastrointestinal disturbances and nephropathy, while for hormonal drugs, efficacy issues, abuse (doping), and carcinogenic potential were reported.

The metabolic, gastrointestinal, and respiratory group includes 20 drugs. Common indications include hypercholesterolemia, type 2 diabetes, obesity, and peptic ulcer disease. Frequently listed drug classes include M_x_R antagonists (*n* = 4), 5-HT_x_R agonists (*n* = 2), and respiratory stimulants (*n* = 2). Adverse effects include gastrointestinal disturbances and metabolic disturbances (including lactic acidosis), cardiac adverse effects (heart attack, arrhythmia, and angina pectoris), hepatotoxicity, and respiratory adverse effects (laryngospasm and infections), among other organ toxicities.

## 6 Observed patterns in discontinued and withdrawn drugs

While every drug removal requires individual consideration, certain characteristics and patterns can be generalized within and between discontinued and withdrawn drugs.

In general, there is a broad variety of drugs, drug classes, and indications associated with market removal. Furthermore, serious adverse effects that led to their removal can be either related to the indication or usage field or completely independent. Examples of related effects include CNS drugs such as benactyzine with psychiatric adverse effects such as hallucinations,^38^ or drugs acting on the cardiovascular system, for example, prenylamine with cardiac adverse effects such as arrhythmia, as well as cytotoxic treatments such as chlornaphazine with carcinogenic effects.^3^^,^^4^^,^^25^ In contrast, effects unrelated to the indication field include the neurotoxicity of diamthazole (antifungal drug), hepatotoxicity in suloctidil (thromboembolic prevention), and carcinogenic potential of danthron (laxative). Such partly predictable and unpredictable adverse effects were already recognized in the analysis of drugs with an FDA boxed warning.^11^ Therefore, target-related effects may be corresponding to the mechanism and therapeutic field, whereas off-target, metabolic, immunologic, or idiosyncratic effects may occur outside the targeted processes.

Both widely prescribed or popular as well as niche or obsolete drugs were found to have been removed. This was observed for both discontinued and withdrawn drugs. Widely prescribed and popular discontinued drugs included doxylamine for nausea in pregnancy (removed following lawsuits concerning suspected fetal malformations), encainide for cardiac arrhythmia (removed because of the induction of ventricular arrhythmia), and thiopental (removed because of political issues regarding its use in executions).^1^^,^^3^^,^^4^^,^^24^^,^^39^ Popular withdrawn drugs included rofecoxib and terfenadine (banned because of cardiac adverse effects), and mibefradil (banned because of significant drug interactions).^1^^,^^3^^,^^4^^,^^25^ Niche or obsolete drug examples include the discontinuation of ethchlorvynol and gallamine (replacement in use by newer treatment options and an unfavorable risk-benefit ratio) and the withdrawal of pentetrazol (second choice in psychiatric treatment, seizures).^4^^,^^40^^,^^41^ For discontinued drugs, the main reason is often obsolescence, with or without safety concerns, combined with no commercial incentive for continued marketing, for example, methicillin, quinethazone, and tridihexethyl.^4^^,^^25^ By contrast, withdrawn drugs are banned because of significant safety or efficacy issues, for example, aminophenazone (agranulocytosis), butamben (transverse myelitis), and trovafloxacin (hepatotoxicity).^3^^,^^4^^,^^25^ Accordingly, the presence of widely used drugs among removals does not alone prove higher intrinsic toxicity. It also reflects greater cumulative exposure and the availability of multiple members within established classes.

Notably, often one specific, serious adverse effect leads to the withdrawal of a drug, for example, hepatotoxicity (pipamazine, trovafloxacin, and troglitazone) or photosensitization of the skin (tribromsalan, dibromsalan, and metabromsalan). This does not mean that a thorough risk-benefit analysis of a drug is absent; rather, a strong safety signal can dominate the assessment when the potential harm is severe and cannot be sufficiently monitored or mitigated. In contrast, the discontinuation of drugs is often multifactorial, with an overall unfavorable risk-benefit ratio (contributing to declining prescriptions), and is based on the consideration of all adverse effects in clinical use. This can also be seen in the assignment of removal-reason categories, with withdrawn drugs often having fewer categories, while discontinued drugs often have a larger number of considered factors. Consequently, regulatory status reflects both the severity and manageability of risks and the route and timing of market removal.

When comparing drug removals with drugs carrying boxed warnings, several shared characteristics are notable, but differences are also apparent. There is a considerable overlap in frequently represented drug classes, major adverse effects, and the variety of adverse effects in relation to the drugs’ usage fields. However, differences are present regarding the regulatory consequences and the market status of the drugs. Consequently, regulatory status reflects both the severity and manageability of pharmacological risk and the route and timing of market removal. A boxed warning permits continued marketing with risk communication, withdrawal reflects a determination that the risk cannot be sufficiently managed for the approved use, and discontinuation may leave approval intact despite the absence of active marketing. Table 2 provides an overview of similarities and differences between US market removals and drugs with an FDA boxed warning.

**Table 2:** Comparison of US market removed drugs with US drugs carrying a boxed warning^11^

Characteristic	US Market Removed Drugs	Drugs With an FDA Boxed Warning^11^
Variety of drugs and indications	Broad variety of drugs, drug classes, and indications	Likewise broad distribution across therapeutic areas
Frequently affected therapeutic areas	Central nervous system, anti-infective drugs, analgesics and immunomodulators, cardiovascular, and renal drugs	Cardiovascular drugs and central nervous system drugs frequently represented
Usage patterns of affected drugs	Both widely prescribed and niche/obsolete drugs affected	Frequently used drugs prominently represented
Frequently represented drug classes	COX inhibitors (*n* = 13), fluoroquinolones (*n* = 10), H1 receptor antagonists, µ-opioid receptor agonists	COX inhibitors and fluoroquinolones also frequently represented
Major adverse effect categories	Hepatotoxicity, cardiac adverse effects, and hematological toxicity are highly prevalent	Cardiac and cardiovascular adverse effects are the most prevalent
Relation between adverse effects and indication	Adverse effects may be related to the indication field or completely unrelated	Similar observation reported
Pattern of adverse effects leading to regulatory action	Often one dominant serious adverse effect leads to withdrawal	Often multiple severe adverse effects lead to the implementation of a boxed warning
Risk-benefit considerations	Withdrawn drugs usually removed because of significant safety or efficacy issues	Boxed warnings indicate serious risks but a remaining positive risk-benefit ratio
Regulatory consequence	Drug discontinuation or withdrawal from the US market	Drug remains approved but carries the strongest FDA safety warning
Market status of affected drugs	Drugs removed from the market or discontinued	Drugs remain available with prominent safety warning

The listed criteria include drug characteristics, prevalent drug classes, and adverse effects, as well as regulatory consequences. Green indicates high similarity, yellow indicates moderate similarity, and orange indicates low similarity.

## 7 Prominent drugs, drug classes, and adverse-effect categories

Certain drug classes comprise multiple removed drugs. Particularly outstanding are COX inhibitors and fluoroquinolones, with 13 and 10 removed drugs, respectively (Fig. 4). Among COX inhibitors, 12 of 13 drugs were removed because of safety issues, and among fluoroquinolones, 8 of 10 were removed for safety reasons (Supplemental Table 1). However, these drugs do not share uniform removal reasons within their drug class, probably because mechanisms of toxicity differ among drugs within the same class. Although they share primary class mechanisms, drug-specific pharmacokinetics and metabolism, off-target activity, interactions, and indications can generate different toxicities. This observation is important because these 2 drug classes are often used uncritically^42^, ^43^, ^44^, ^45^ and are perceived as “safe” medications.^46^^,^^47^ Their widely established use may partly explain the large number of withdrawals because many drugs from each class were marketed, with some of them displaying more unfavorable characteristics than others. This shows the importance of differences between drugs of the same drug class and also highlights a precautious use of the drug classes themselves,^48^^,^^49^ with both fluoroquinolones and COX inhibitors carrying boxed warnings from the FDA and being an outstanding prevalent drug class there too.^11^

Regarding adverse effects contributing mainly to drug removal, hepatotoxicity and cardiac and hematologic adverse effects are highly prevalent (Fig. 3; Supplemental Table 3). Hepatotoxicity may occur frequently because of the central role of the liver in (drug) metabolism and the potential for toxic interference with multiple signaling pathways.^50^, ^51^, ^52^, ^53^ The cardiovascular system and its electrophysiology are highly sensitive to physiological changes,^54^ and the hematologic system with its rapidly renewing cells can be quickly affected by drug toxicity.^55^^,^^56^ Cardiac and cardiovascular adverse effects were also found to be the most prevalent drug class in boxed warnings.^11^ These organ systems therefore combine biological susceptibility with high regulatory relevance because injury can be severe, difficult to predict, or rapidly consequential. Their recurrence in both removals and boxed warnings indicates a continuum of regulatory responses, from risk communication and monitoring to complete market withdrawal, depending on whether the risk can be managed relative to the therapeutic benefit.

Some drugs were observed to re-enter the market several years after their removal, including alosetron, fenfluramine, tegaserod, and doxylamine. Fenfluramine, originally approved as an antiobesity drug, was withdrawn in 1997 because of cardiac fibrosis. Since 2024, it has been approved again for another condition, as an antiseizure drug for Dravet syndrome.^3^^,^^4^ Tegaserod and alosetron, used for irritable bowel syndrome, were voluntarily discontinued, alosetron because of serious gastrointestinal adverse events (ischemic colitis and perforations) in 2000 and tegaserod because of cardiac adverse effects (heart attack, stroke, and unstable angina pectoris) in 2007. However, both were reapproved for the same condition, but being only indicated for women.^4^^,^^25^ Doxylamine, used for insomnia, allergic symptoms, and the treatment of nausea in pregnancy, was discontinued in 1983 in relation to lawsuits about fetal malformations. This relationship was later disproved, and the drug was reinstated in 2013 as an over-the-counter drug.^4^^,^^39^^,^^57^

These 3 examples highlight that the market removal of drugs is not absolute and can be changed by scientific research, repurposing, and re-evaluation of the risk-benefit ratio. They also show that regulatory decisions are indication- and context-specific rather than a permanent judgment of a drug. Changes in the indication, patient population, monitoring, or therapeutic need can shift the risk-benefit balance. Reapproval does not erase the previous safety concerns but narrows the conditions under which its use is considered acceptable. Table 3 provides a detailed overview of the re-entered drugs.

**Table 3:** Overview about drugs that re-entered the US market after drug removal

Drug	Initial Indication	Removal Y	Reason for Removal	Reintroduction Y	Indication After Reintroduction	Notes	Reference
Doxylamine	Insomnia, allergic symptoms, nausea in pregnancy	1983	Lawsuits concerning fetal malformations (discontinued)	2013	Insomnia, allergic symptoms, nausea in pregnancy (OTC drug)	Reintroduced after reassessment of safety for similar indications	^2^^,^^4^^,^^5^^,^^25^^,^^39^^,^^57^
Fenfluramine	Antiobesity treatment	1997	Cardiac fibrosis (withdrawn)	2024	Dravet syndrome	Reintroduced for a different indication	2, 3, 4, 5^,^^25^
Alosetron	Irritable bowel syndrome	2000	Ischemic colitis, gastrointestinal perforations (discontinued)	2002	Irritable bowel syndrome (women only)	Restricted indication	^2^^,^^4^^,^^5^^,^^25^
Tegaserod	Irritable bowel syndrome	2007	Cardiac adverse effects (discontinued)	2019	Irritable bowel syndrome (women only)	Restricted indication	^2^^,^^4^^,^^5^^,^^25^

Given is the initial and the current indication, the y of removal and reintroduction, the primary reason for removal, and an explanation of changes in use connected with the reintroduction. Further details can be found in Supplemental Tables 1 and 2. Drugs are sorted by the y of their market removal.

## 8 Comparison of drugs removed from US market with the EU market

To provide a further assessment and an international comparison, the drugs removed from the US market were compared with the EU market. While the FDA is solely responsible for the entire US market, pharmaceutical regulation in the EU involves individual countries with their own national authorities, coordinated by the European Medicines Agency (EMA) since 1995.^58^, ^59^, ^60^ For the comparison, drugs removed from the US market were searched for the medicines data table of centrally authorized medicines,^61^ and the Article 57 data base covering medicines authorized in the European Economic Area.^62^

A total of 130 US removed drugs were not listed in any of the considered data bases of approved or withdrawn drugs maintained by the EMA or European national authorities. This indicates that the drugs were either never centrally authorized by the EMA, or are no longer approved by individual European countries. Nine drugs were withdrawn by the EMA, for 1 drug (tegaserod) the approval was refused, and for 1 drug (telaprevir), the EU marketing authorization expired.

Twenty-seven drugs are approved and marketed in at least one EU country. Of these, 25 drugs were removed from the US market, including 15 of them withdrawn by the FDA. In addition, there are 2 drugs (tegaserod and alosetron) that were reintroduced in the US market for irritable bowel syndrome in women, but are not approved in any EU member country. Supplemental Table 4 provides an overview of the status in the EU of drugs removed from the US market, and Fig. 5 visualizes similarities and differences in market authorization.

**Fig. 5:**
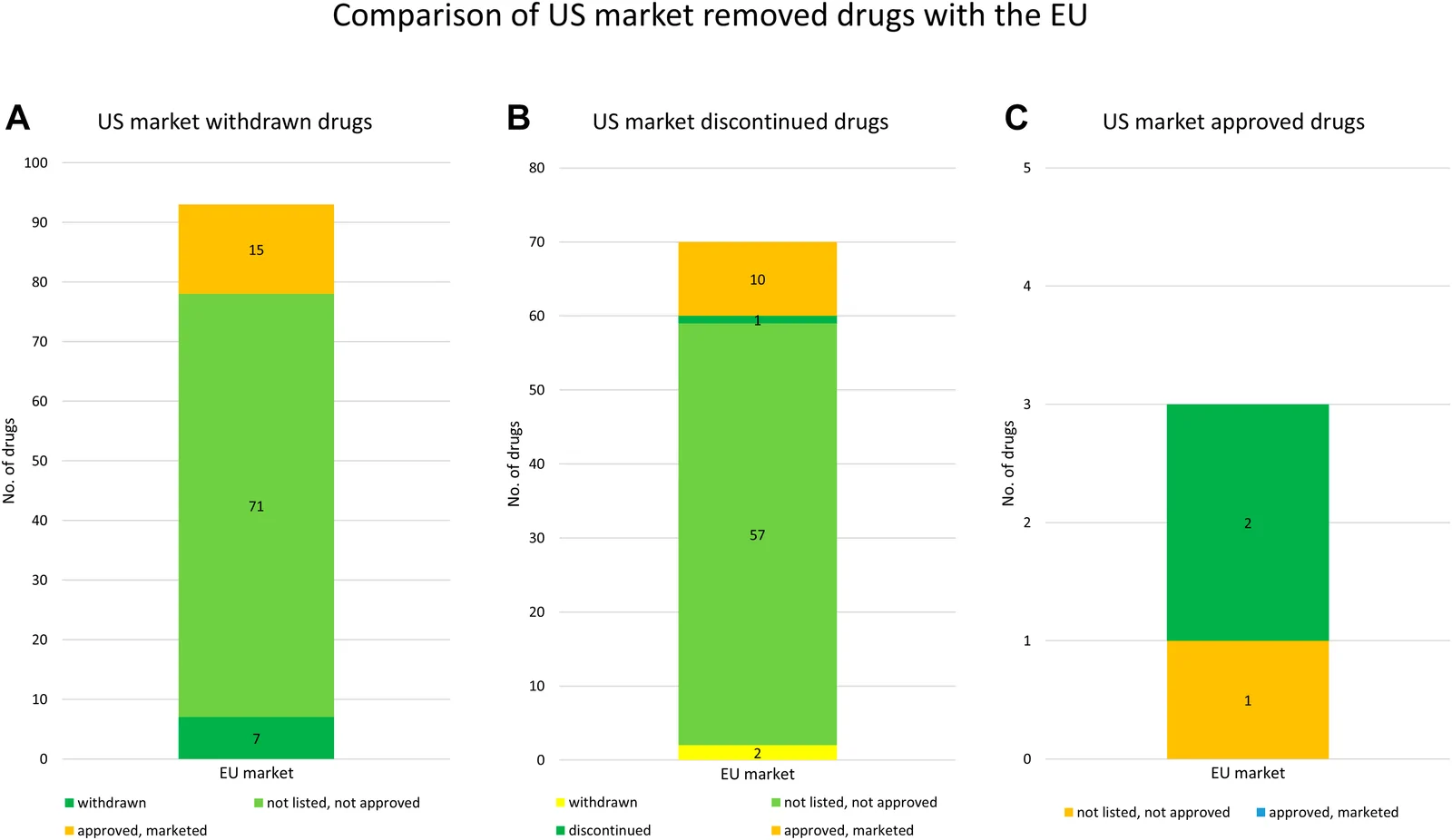
Comparison of the analyzed drugs that were removed from the US market with their status in the EU. Depicted are US drugs that were (a) withdrawn by the FDA, (b) discontinued by the manufacturer, and (c) once withdrawn but later remarketed. Green highlights consistency or similarity between the market and legal status in the United States and the EU, yellow indicates drugs that are consistent in market status but differ in their legal status, and orange represents drugs that differ between the EU and the US market.

Comparisons with the literature confirm differences in the regulatory procedures and decisions of the FDA and EMA/EU,^63^^,^^64^ which can be explained by their varying organizational structures, responsibilities, and priorities in pharmaceutical regulation.^13^^,^^65^ The observed differences highlight cultural and country-specific variation in the risk-benefit assessment and drug safety evaluation in international regulatory contexts.^13^^,^^66^, ^67^, ^68^

## 9 Publication peaks of removed drugs

A PubMed^69^ search was conducted on March 20, 2026, for the substance name for each drug. It was possible to analyze the publication trend for 163 of the 166 removed drugs. For 3 drugs, it was not possible to distinguish the use as a medication from other investigations, including tryptophan and adrenal cortex, while for cinoxacin, no literature results were found. For 159 of the 166 drugs, it was possible to calculate the delay between the market removal and the publication peak, while 7 drugs lacked data on either the market removal date or publication peak (Supplemental Table 5). The time course of publication peaks is visualized in Supplemental Fig. 1.

The majority of drugs, 137 of 163, had 1 publication peak. Two publication peaks were found for 17 drugs, 3 for 6 drugs, and 4 for 2 drugs. For most drugs, 100, their publication peak occurred before market removal, while for 39 drugs, it occurred after the market removal. For 14 drugs, the publication peak occurred in the same year as their removal, while it for chlormadinone and rapacuronium it occurred before or in the same year as removal because either 2 removal dates or 2 publication-peak dates were identified. For 3 drugs, potassium arsenite, mepazine hydrochloride, and hydrocortamate, publication peaks were unspecific, meaning that they occurred both before and after the market removal (Fig. 6).

**Fig. 6:**
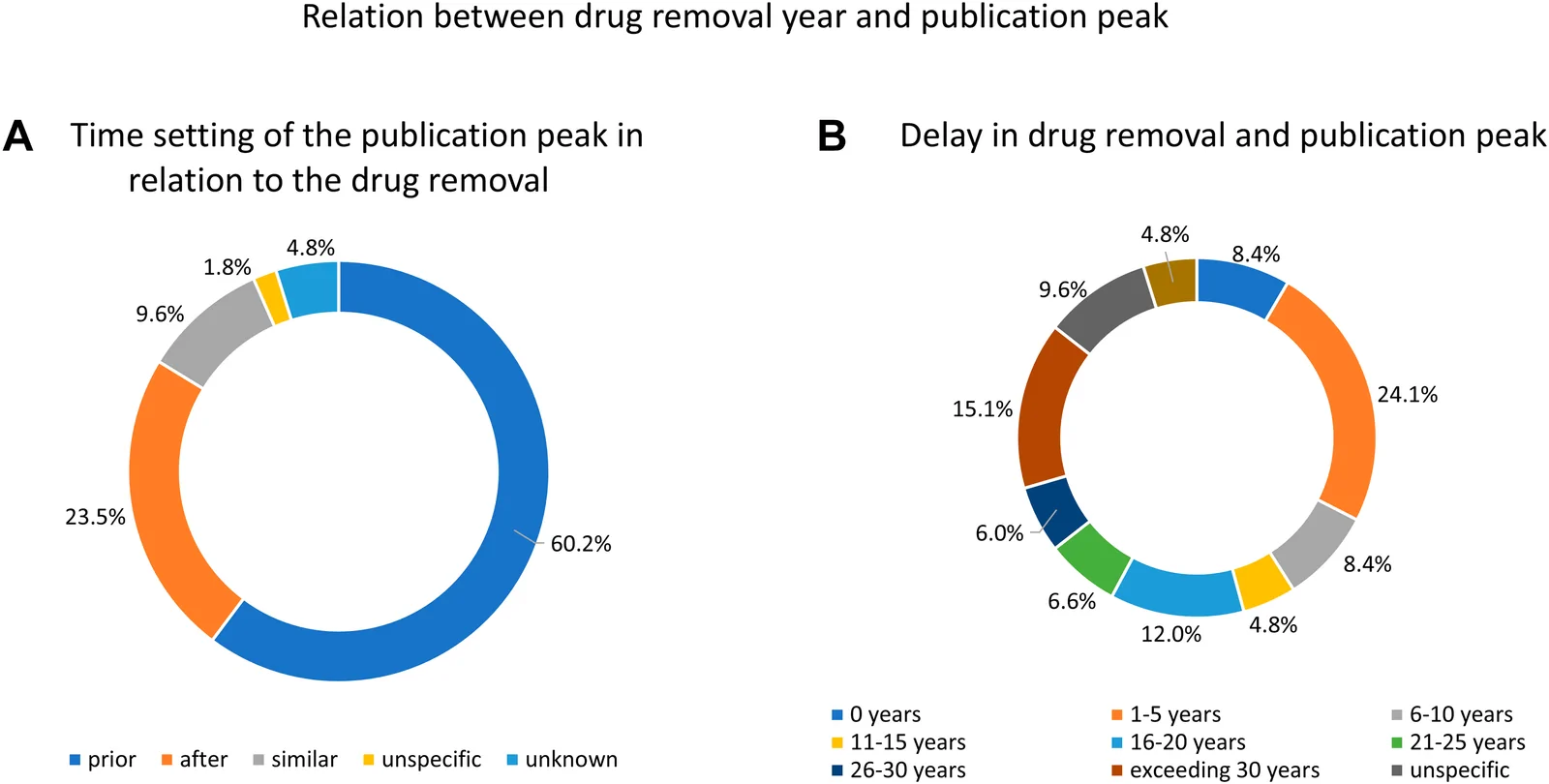
Connection between the year of the drug removal and its publication peak. In (a) the time setting of the publication peak in relation to the drug removal is depicted, while (b) provides an overview about the delay in years between both events. For each category, the share of the drugs on the total number of removed drugs is given. The category “unspecific” includes drugs that had multiple publication peaks, both before and after the drug removal. In the category “unknown,” the relation cannot be stated because either the year of the drug removal is uncertain or it has not been possible to conduct a search on PubMed with the specific use of the substance as a therapeutic drug.

The delay between the drug market removal and the publication peak varied considerably, from 0 to 60 years. While 49 drugs had a delay of ≤5 years, the delay exceeded 20 years for 41 drugs (counted for drugs with a single publication peak) (Fig. 6). Among drugs with multiple publication peaks, the delay could be short (eg, chlormadinone and rapacuronium, 0–2 years), very long (eg, tridihexethyl, 48–49 years), or vary markedly depending on the publication peak (eg, metabromsalan, 6–44 years). For drugs with a single publication peak, the mean delay was 15.4 years.

## 10 Relationship between the drug removal year and its publication peak

In addition to information about the peak of scientific interest in a drug and its relation to its removal, 3 recurring patterns can be observed that relate the timing of publication peaks to the regulatory status and market removal of a drug. These observed patterns were systematized to allow a structured characterization of removed drugs. They may serve as a screening tool to identify changes in scientific interest that could potentially influence future regulatory decisions or therapeutic reconsideration. The systematized pattern characteristics are summarized in Table 4, while Fig. 7 illustrates the 3 patterns using the exemplary drugs levallorphan, rimonabant, and dydrogesterone as examples. Because publication counts measure scientific attention rather than prescriptions or therapeutic importance, these patterns should not be interpreted as direct evidence of drug use.

**Table 4:** Overview about systematized patterns relating publication peaks to drug market removal

Pattern	Status	Temporal Relation Between Publication Peak and Market Removal	Interpretation	Exemplary Drugs
a – Declining scientific interest	Discontinued	Publication peak occurs before market removal, often many y before (large delay)	Indicates declining relevance and clinical use; removal often associated with obsolescence or commercial decisions	Levallorphan, clofibrate, dicumarol
b – Safety-driven scientific attention	Withdrawn	Publication peak occurs in the same y as or close to market removal	Reflects intensified scientific discussion triggered by safety concerns leading to regulatory withdrawal	Cisapride, rimonabant, flosequinan, troglitazone, zimelidine
c – Renewed scientific interest	Discontinued or withdrawn	Publication peak occurs after market removal, often in recent y	Suggests renewed investigation, potential repurposing, or re-evaluation of the drug	Thiabendazole, dydrogesterone

Given is the typical legal status of the drug (discontinued or withdrawn), the temporal relation between the publication peak and the y of market removal, the interpretation of the observed pattern, and representative example drugs. The patterns correspond to the exemplary drugs depicted in Fig. 7. Information for each drug about its characteristics regarding its publication peak can be found in the Supplemental Table 5.

**Fig. 7:**
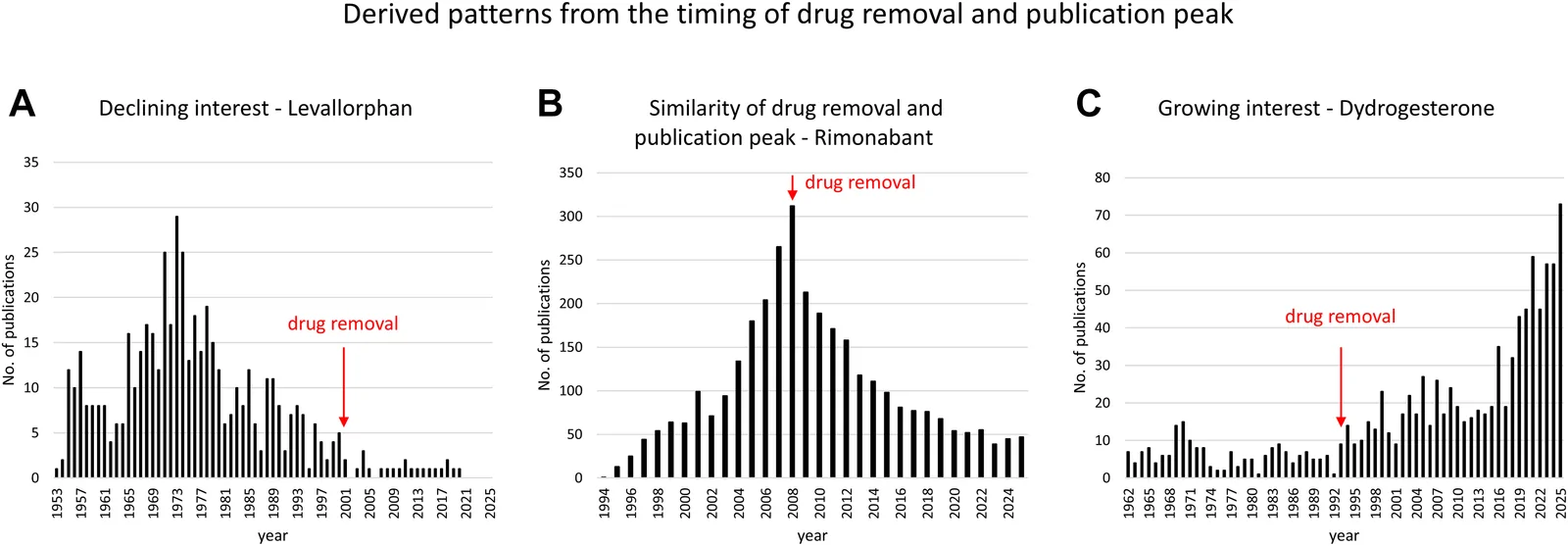
Derived patterns from the relation of the publication peak to the year and cause of the drug removal. Each pattern is shown for a prototypical drug, with its number of publications from each year derived from PubMed, from the year of the first publication until 2025. In (a), a decreasing interest, consisting of a discontinued drug because of commercial reasons and a publication peak before the removal is depicted. In (b), the drug removal and the publication peak occur in the same year, suggesting the highest attention due to the drug’s removal. In (c), the combination of an increasing interest in the last years, occurring after the drugs removal, suggests a growing scientific interest.

A first pattern suggests declining scientific interest, with the drug becoming obsolete and losing importance. It is characterized by a discontinued legal status and a publication peak before the market removal. The longer the delay in years, the stronger the expression of the pattern. Because the manufacturer removed the drug voluntarily, and the scientific interest peaked considerably earlier, this suggests a declining use, and a removal for commercial reasons and obsolescence. Examples of this pattern are levallorphan, clofibrate, and dicumarol, all discontinued because of business decisions after being replaced in use by other treatment options.^4^^,^^25^ The inference is strongest when declining publications coincide with known replacement or declining prescriptions, while the publication peak alone cannot verify market use.

The second observed pattern combines a withdrawn drug with a publication peak around the time of removal. The publication peak likely reflects the scientific discussion and safety-driven attention around the market removal rather than continued therapeutic use. Examples are cisapride, rimonabant, flosequinan, troglitazone, and zimelidine, all withdrawn because of safety reasons, with a publication peak in the same year as the market ban.^1^^,^^3^^,^^4^^,^^27^

A third pattern may indicate increasing relevance and growing scientific interest. It combines a discontinued or withdrawn drug with a recent publication peak after its removal and growing publication trend. This may be due to increasing scientific research, for example, a re-evaluation or drug repurposing. Examples are thiabendazole investigated in cancer treatment^70^^,^^71^ and dydrogesterone in gynecology.^72^ However, evaluation based on a recent publication peak can be strongly distorted by a low number of publications because a single recent publication can create a misleading conclusion. Moreover, postremoval interest may indicate scientific repurposing without corresponding market demand or impending reapproval. Nevertheless, these patterns remain valuable as a screening tool.

## 11 Conclusions and future directions

The analysis of drugs removed from the US market revealed a diverse group of drugs, indications, and causes (Supplemental Tables 1–3). A distinction can be made between drugs voluntarily discontinued by the manufacturer and those withdrawn by the FDA (Table 1). While manufacturer decisions to remove drugs can be based on business decisions or commercial grounds as well as safety or efficacy issues, the FDA bans drugs because of serious safety or efficacy issues and does not consider commercial grounds. However, there is a gradual transition between manufacturer decisions based on commercial grounds and those related to safety or efficacy considerations, as well as between the voluntary discontinuation of drugs with safety issues and the withdrawal by the FDA. Therefore, the 2 legal statuses are distinct regulatory outcomes but not distinct pharmacological decisions: they may include similar toxicities while differing in who initiated removal and whether approval remains in place.

Notably, many removed drugs belong to established and frequently used drug classes, such as COX inhibitors, MOR agonists, histamine receptor 1 antagonists, or fluoroquinolones (Fig. 4). Some drugs within these drug classes are available as over-the-counter, with a tendency toward overuse, and are often (mis-)perceived as “safe” medications. While one might argue that many drug preparations were developed for the respective drug classes and that of them have an unfavorable drug profile, it remains notable that drugs for serious conditions (eg, cancer treatment) are not listed in such high numbers. This observation suggests that market withdrawals are not necessarily concentrated in areas with the highest therapeutic risk. The contrast illustrates how therapeutic context modifies regulatory assessment. In common conditions with numerous alternatives, even an uncommon serious toxicity can make one drug replaceable. In life-threatening conditions, greater risk may remain acceptable when the benefit is substantial and alternatives are limited. At the same time, high use increases exposure and the opportunity to detect rare harms. Removal counts therefore reflect pharmacology, prescription volume, available alternatives, and regulatory tolerance rather than toxicity alone.

Assessment of the safety profile and the risk-benefit ratio of a drug is not entirely objective. Furthermore, the corresponding decisions are neither generalizable to the entire drug class nor absolute, as shown by the reapproval of drugs removed for safety reasons. Either for the same indication (eg, tegaserod), or for another condition (eg, fenfluramine), for which the drug is considered favorable under specific conditions. Other important considerations relate to country-specific and cultural differences. While some drugs are banned from the US market, they are available in other markets (eg, metamizole), and vice versa. In this context, the assessment of publication peaks may indicate whether a drug is experiencing renewed interest and a reinvestigation (Fig. 7; Table 3 and Supplemental Table 3), which may lead to potential therapeutic use and future remarketing. However, scientific attention alone does not establish renewed clinical use or a favorable regulatory reassessment.

Apart from the unfavorable causes that led to drug removal, there can also result positive aspects. Discontinuation for commercial reasons often concerns drugs that have become obsolete or unfavorable compared with other treatment options, thereby supporting rational pharmacological treatment through the removal of inferior options. At the same time, the ongoing pharmacovigilance, drug-safety monitoring, and assessment of the risk-benefit ratio by the FDA contribute strongly to pharmacotherapy safety by banning drugs that cause more harm than benefit. Together, these mechanisms guide treatment decisions, protect patients, and contribute to better treatment outcomes.

In general, there is a strong need for a comprehensive overview of drugs withdrawn for safety reasons from the US market. Existing governmental declarations, for example, §216.24 of the Federal Food, Drug, and Cosmetic Act, have not been updated since 2018 and are therefore outdated. Available data bases such as Inxight Drugs, DrugBank, or PubChem^4^^,^^69^^,^^73^ are incomplete, with information fragmented across them. Other data base collection projects such as Withdrawn 2.0 are a good start but also include incorrect and incomplete information. In general, information is often sparse and must be collected from various references, with some information unavailable (eg, exact dates of withdrawal or discontinuation by all manufacturers). Online-accessible drug safety information provided by the FDA is incomprehensive and primarily focuses on drug preparations instead of the general safety of a drug. Table 5 provides an overview of available sources, along with an assessment of their value and restrictions. A regularly updated, comprehensive overview, preferably with explanations for market removal, for example, in a separate section of the Orange Book, would be very valuable for both scientific research and clinical practice, thereby improving drug safety.

**Table 5:** Summary of included references that were used in the identification and assessment of drugs removed from the United States and EU market

Data Source	Use in the Analysis	Value	Restrictions/Incompleteness
FDA Orange Book^2^	Verification of US approval status; differentiation of active and discontinued products	Official FDA data; regular updates; product-level status; discontinued drug product list	Focus on preparations/applications rather than substance; withdrawn approvals absent; removal reasons and exact dates often unavailable; incomplete for historical drugs (before 1980) since time frame cutoff, corresponding to the first edition in 1980
Withdrawn 2.0^5^	Initial collection of withdrawn drugs; safety and pharmacological information	Broad, structured, searchable dataset; useful starting point; international applicability; information about legal status globally; link to primary literature	Incomplete or incorrect entries; not US-specific; unclear differentiation between withdrawal and discontinuation; missing dates and reasons
Inxight Drugs^4^	Verification of status, indications, mechanisms, and synonyms	Integrated pharmacological and regulatory information; broad drug coverage; up-to-date	Fragmented market histories; mixed information across countries, indications, and preparations; sparse removal reasons and dates
PubChem^73^	Drug identifiers, synonyms, structures, and safety information	Comprehensive chemical information; public accessibility; extensive database links	No systematic regulatory history; incomplete withdrawal information; ambiguity between drugs, salts, and preparations
DrugBank^25^	Mechanisms, targets, indications, and drug-class allocation	Curated pharmacological profiles; structured mechanism and target information summarized from multiple sources	Incomplete regulatory timelines; secondary market-status information; country- and preparation-specific differences
US Government §216.24^9^	Identification of products withdrawn for safety or efficacy reasons	Formal legal source; explicit regulatory basis; information about reasons	Last update in 2018; missing recent withdrawals; product-specific information; manufacturer discontinuations excluded
FDA Drug Safety Communications (multiple)	Identification and evaluation of recent safety actions	Official and recent safety information; regulatory explanations and timelines	Selected communications only; no comprehensive historical overview; safety communication not equivalent to formal withdrawal
Bindel and Seifert^11^	FDA boxed-warning dataset; comparison of drug classes and adverse-effect categories	Structured safety comparison; comprehensive and current (April 2026) overview of boxed warnings; availability of reasons; identification of recurring pharmacological patterns	Changing warning status over time; no updates in regard to time frame; indication- and preparation-specific warnings; no direct information on market removal
EMA databases/Article 57^61^^,^^62^	Comparison of US removals with EU market status	Official EU/EEA authorization data; centralized and national product coverage	Heterogeneous national information with potential differences in legal status in EU member countries; incomplete historical coverage (start since 1995); limited withdrawal reasons and dates; preparation-specific differences
PubMed^69^	Publication-count data; identification of publication peaks and scientific-interest patterns	Longitudinal and reproducible indicator of scientific attention; inclusion of primary literature without restricted time frame	No direct measure of consumption or therapeutic importance; drug-name ambiguity; distortion by low publication numbers; safety-related and scientific interest not distinguishable

For each data source, its value vs its restriction is discussed, along with information on how the data was used in the analysis.

EEA, European Economic Area.

Further research is needed to evaluate market removals and their reasons in other countries, for example, in the EU, and compare them with those in the US market. This would help to explain country-specific differences in drug risk-benefit assessments. Furthermore, a thorough assessment of the consequences of discontinued and withdrawn drugs in other markets would be informative, as would an analysis of past and emerging therapeutic investigations of drugs removed from the US market. However, scientific attention alone does not establish renewed clinical use or a favorable regulatory reassessment. Future analyses should integrate prescription volume, duration of market availability, use across indications, and the timing of safety communications with pharmacological mechanisms and regulatory outcomes. This would help distinguish class-specific safety patterns from high removal counts driven by extensive use or a large number of marketed preparations.

## Conflict of interest

The authors declare no conflicts of interest.
