# Supplementary material for: US postmarketed drug removals: Analysis of discontinued and withdrawn drugs
Source: J Pharmacol Exp Ther. 2026 Jul 31;393(9):104996. doi: 10.1016/j.jpet.2026.104996 (PMC13688983; doi:10.1016/j.jpet.2026.104996)
Supplement: Supplementary file 1 [file mmc1.docx]

**US post-marketed drug removals:**

**analysis of discontinued and withdrawn drugs – Supplemental Files**

**Lilly Josephine Bindel^1^ and Roland Seifert^1^**

**^1^ Institute of Pharmacology, Hannover Medical School, D-30625 Hannover, Germany
Corresponding author: Roland Seifert, seifert.roland@mh-hannover.de**

Supplemental Figures

***Supplemental Figure 1:*** *Time course of publication peaks of removed drugs. On the x-axis, the year is given chronologically, while the y-axis depicts the number of drugs. The bar charts (blue-colored) give the number of drugs with a publication peak, while the dots (orange-colored) give the number of drug removals. Drugs were listed once for their year of removal, while multiple listings of publication peaks within a single drug are included.*

Supplemental Tables

***Supplemental Table 1:*** *Overview about drug withdrawals from the US market. Given is the drugs name, its removal year (unknown dates stated as “?”), its status, its primary removal cause, its indication, and drug class. Drug removals that were initially discontinued by the manufacturer but later withdrawn by the FDA are listed under withdrawn drugs. Drugs are sorted alphabetically.*

| **Drug Name (synonym)** | **Drug class** | **Formerly FDA-approved indication** | **Year of removal** | **Status** | **Removal category** | **Reason of market removal [1, 3-5, 9, 19, 21]** |
| --- | --- | --- | --- | --- | --- | --- |
| chlormadinone | progesterone receptor agonist | anti-estrogenic, contraceptive | 1970, 1972 | discontinued, withdrawn | safety | carcinogenic (development of mammary tumors in dogs) |
| methoxyflurane | synaptic transmission inhibitor | inhalational anesthetic (analgesic, muscle relaxation) | 2001, 2005 | discontinued, withdrawn | safety | safety concerns, nephrotoxic and hepatotoxic |
| nomifensine | MAO inhibitor | depression | 1986, 1992 | discontinued, withdrawn | safety | hemolytic anemia, hypersensitivity reactions, hepatotoxicity, thrombocytopenia |
| novobiocin | fluoroquinolone, DNA gyrase inhibitor | antibacterial drug | 1999, 2011 | discontinued, withdrawn | manufacturer choice, safety | development of safer and more effective drugs, skin reactions, jaundice, hepatic failure, blood dyscrasis (neutropenia, anemia, thrombocytopenia), development of resistant *staphylococci*, potential drug interactions |
| pemoline | Dopamine agonist (mechanism unknown) | ADHD, narcolepsy | 2005 | discontinued, withdrawn | manufacturer choice, safety | economic reasons, liver toxicity and other adverse effects (aplastic anemia, convulsive seizures, hallucinations, insomnia, anorexia) |
| temafloxacin | fluoroquinolone, DNA gyrase inhibitor | antibacterial drug | 1992, 1997 | discontinued, withdrawn | safety | hypoglycemia, hemolytic anemia, kidney and liver dysfunction, allergic reactions, coagulopathy |
| ticrynafen (tienilic acid) | NKCC inhibitor | loop diuretic, hypertension | 1980, 1996 | discontinued, withdrawn | safety | hepatotoxicity |
| ***Drug Name (synonym)*** | ***Drug class*** | ***Formerly FDA-approved indication*** | ***Year of removal*** | ***Status*** | ***Removal category*** | ***Reason of market removal [1, 3-5, 9, 19, 21]*** |
| sulfathiazole | sulfonamide, dihydropteroate synthase inhibitor | antibacterial drug | 1970 | discontinued/ withdrawn | safety | renal complications, rash, fever, blood dyscrasias, liver damage |
| adenosine phosphate | 5'-AMP-activated protein kinase | cardiac arrhythmia | 1973 | withdrawn | safety | cardiac adverse effects (high degree heart block, cardiac arrest, ventricular arrhythmia, myocardial infarction), not safe |
| adrenal cortex | GCR agonists | hormone substitution, inflammatory conditions | 1978 | withdrawn | efficacy, safety | substantial risk of undertreatment due low level of GCR agonists; significant potential hazard |
| alphacetyl-methadol | MOR agonist | maintenance treatment of opioid dependence | 2003 | withdrawn | safety | cardiac adverse effects (ventricular arrhythmias, torsade de pointes) |
| alpha-ethyltryptamine (etryptamine) | MAO inhibitor | depression | 1962 | withdrawn | safety | abuse, dependence, agranulocytosis |
| althesin | GABA-modulator | anesthetic | 1984 | withdrawn | safety | anaphylactic reactions |
| aminopyrine (amino-phenazone) | pyrazolone | analgesic drug, antipyretic drug | 2000 | withdrawn | safety | agranulocytosis |
| aprotinin | serine protease inhibitor | antifibrinolytic drug | 2007 | withdrawn | safety | increased mortality (clinical trial) |
| azaribine | pyrimidine analogon, antimetabolite | psoriasis | 1977 | withdrawn | safety | thromboembolic events |
| benactyzine | AChE inhibitor | depression, anxiety | ? | withdrawn | safety | vegetative and CNS adverse effects (anticholinergic syndrome, hallucinogenic) |
| ***Drug Name (synonym)*** | ***Drug class*** | ***Formerly FDA-approved indication*** | ***Year of removal*** | ***Status*** | ***Removal category*** | ***Reason of market removal [1, 3-5, 9, 19, 21]*** |
| bithionol | soluble adenylyl cyclase inhibitor | antibacterial, anthelmintic, algaecide | 1967 | withdrawn | safety | photosensitization, causing serious skin disorders |
| bunamiodyl | contrast agent | cholecystographic diagnostic drug | 1963 | withdrawn | safety | nephropathy |
| butamben | voltage-gated calcium channel inhibitor | local anesthetic | 1964 | withdrawn | safety | severe tissue slough, transverse myelitis |
| chloramphenicol | bacterial ribosome inhibitor | antibacterial drug | 2011 | withdrawn | safety | induction of aplastic anemia |
| chlornaphazine | naphthylamine derivative | Hodgkins' disease | 1964 | withdrawn | safety | carcinogenic (bladder tumors) |
| chloroform | GABA-A receptor modulator | anesthetic | 1976 | withdrawn | safety | carcinogenic (animal tests) |
| cisapride | 5-HT_4_ receptor agonist | nocturnal heartburn due to GORD | 2000 | withdrawn | safety | fatal arrhythmia |
| danthron | anthraquinone | laxative | 1987 | withdrawn | safety | carcinogenic |
| dexfenfluramine | SSRI | obesity | 1997 | withdrawn | safety | association with valvular heart disease |
| dextropropoxyphene | MOR agonist | pain, antitussive, local anesthetic | 2010 | withdrawn | safety | risk of cardiac arrhythmias and overdose |
| diamthazole | antifungal drug | tinea pedis | 1972 | withdrawn | safety | neuropsychiatric adverse reactions, neurotoxicity |
| dibromsalan | halogenated salicylanilides | antimicrobial | 1975 | withdrawn | safety | photosensitizer, disabling skin disorders |
| ***Drug Name (synonym)*** | ***Drug class*** | ***Formerly FDA-approved indication*** | ***Year of removal*** | ***Status*** | ***Removal category*** | ***Reason of market removal [1, 3-5, 9, 19, 21]*** |
| dihydrostreptomycin | bacterial ribosome inhibitor | antibacterial drugs | 1970 | withdrawn | safety | ototoxicity, higher risk of deafness without greater effectiveness than streptomycin |
| dimethylamyl-amine | amphetamine derivative | allergic/infectious rhinitis | 1983 | withdrawn | safety | cardiovascular adverse effects (vasoconstriction, hypertension, myocardial infarction), neuropsychiatric adverse effects, seizures |
| dipyrone (metamizole) | pyrazolone derivative | analgesic, antispasmodic, antipyretic | 1977 | withdrawn | safety | agranulocytosis |
| dithiazanine iodide | benzothiazole | anthelminthic drug | 1964 | withdrawn | safety | highly toxic (fatal cases of severe acidosis and shock) |
| drotrecogin alfa | coagulation factor V and VIII inhibitor | reduction of mortality in severe sepsis | 2011 | withdrawn | efficacy | not effective in improving outcomes in septic patients |
| etamivan | respiratory stimulant, methoxyphenol | pulmonary emphysema | ? | withdrawn | safety | coughing, laryngospasm, seizures |
| ethoheptazine | MOR agonist (uncertain) | analgesic (pain, headache, musculoskeletal disorders) | 1984 | withdrawn | manufacturer choice, safety, efficacy | business decision, toxicity and efficacy issues (not specified), replaced by benzodiazepines |
| ethyl biscoumacetate | VKA | anticoagulant, thromboembolic disorders | 1999 | withdrawn | safety | severe bleeding, narrow therapeutic index |
| ethyl nitrite (sweet spirits of nitre) | NO donor | cough, flu (symptomatic treatment) | 1980 | withdrawn | safety | methemoglobinemia in infants |
| ***Drug Name (synonym)*** | ***Drug class*** | ***Formerly FDA-approved indication*** | ***Year of removal*** | ***Status*** | ***Removal category*** | ***Reason of market removal [1, 3-5, 9, 19, 21]*** |
| fenclozic acid | COX inhibitor | analgesic, antispasmodic, antipyretic | 1970 | withdrawn | safety | jaundice, hepatotoxicity |
| flosequinan | PDE_3_ inhibitor | heart failure, vasodilator | 1993 | withdrawn | safety, efficacy | increased mortality and hospitalization rates; no long-term benefit; increase of congestive hart failure symptoms |
| glafenine | COX inhibitor | analgesic, antispasmodic, antipyretic | 1992 | withdrawn | safety | anaphylaxis, acute kidney failure |
| indoprofen | COX inhibitor | analgesic, antipyretic, anti-inflammatory | 1984 | withdrawn | safety | severe gastrointestinal bleeding and perforation, carcinogenic |
| iproniazid | MAO inhibitor | depression | 1961 | withdrawn | safety | hepatotoxicity |
| levamisole | nicotinic ACh agonist | anthelmintic; colorectal carcinoma; rheumatoid arthritis | 2000 | withdrawn | safety | serious adverse effects, e.g. agranulocytosis |
| mepazine hydrochloride | MALT protease inhibitor | antineoplastic drug, MALT | 1970 | withdrawn | safety | granulocytopenia, agranulocytosis, paralytic ileus, urinary retention, seizures, hypotension, jaundice |
| metabromsalan | salicylanilide | antimicrobial, pesticide | 1975 | withdrawn | safety | photosensitizer, disabling skin disorders |
| metandienone (methandrostenolone) | androgen receptor agonist | post-menopausal osteoporosis, pituitary-deficient dwarfism | 1982 | withdrawn | safety | misuse and substance abuse (sports) |
| methaqualone | GABA agonist | sedative, anxiolytic | 1982 | withdrawn | safety | abuse, misuse, dependence |
| methopholine | MOR agonist | analgesic | 1965 | withdrawn | safety | ophthalmic changes and corneal opacities in dogs |
| ***Drug Name (synonym)*** | ***Drug class*** | ***Formerly FDA-approved indication*** | ***Year of removal*** | ***Status*** | ***Removal category*** | ***Reason of market removal [1, 3-5, 9, 19, 21]*** |
| muzolimine | NKCC inhibitor | loop diuretic | 1987 | withdrawn | safety | severe neurological effects |
| nikethamide | respiratory stimulant | countermeasure against tranquilizer overdoses | 1988 | withdrawn | safety | toxicity, misuse, banned doping substance |
| oxeladin | centrally acting medullary cough depressant | cough suppressant | 1976 | withdrawn | safety | carcinogenic |
| oxyphenisatin, oxyphenisation acetate | peristalsis stimulant | enema for diagnostic and operative procedures | 1972 | withdrawn | safety | hepatitis, jaundice |
| parecoxib | COX inhibitor | rheumatic disorders (osteoarthritis, adult rheumatoid arthritis), primary dysmenorrhea | 2005 | withdrawn | safety | unfavorable risk-benefit ratio, higher risk of cardiovascular adverse effects and skin reactions (toxic epidermal necrolysis, Stevens-Johnson syndrome, erythema multiforme) compared with other COX inhibitors no advantages |
| pentetrazol (pentylen-etetrazol) | GABA antagonist | respiratory depression, idiopathic hypersomnia, induction of seizures in schizophrenia treatment | 1982 | withdrawn | safety, efficacy | seizures, availability of mGPCR antagonists and electroconvulsive therapy for schizophrenia treatment |
| pergolide | Dopamine receptor agonist | Parkinson's Disease | 2007 | withdrawn | safety | heart attack, stroke, unstable angina pectoris, increased risk of cardiac valvopathy |
| phenacetin | COX inhibitor | analgesic, antipyretic | 1983 | withdrawn | safety | methemoglobinemia, carcinogenic (renal pelvis, ureter) |
| phenformin | biguanide | diabetes type 2 | 1978 | withdrawn | safety | high incidence of lactic acidosis |
| ***Drug Name (synonym)*** | ***Drug class*** | ***Formerly FDA-approved indication*** | ***Year of removal*** | ***Status*** | ***Removal category*** | ***Reason of market removal [1, 3-5, 9, 19, 21]*** |
| phenolphthalein | efflux of potassium from cells | laxative | 1999 | withdrawn | safety | carcinogenic |
| phenylpropanolamine | α-AR agonist | nasal decongestant, anorexiant | 2000 | withdrawn | safety | hemorrhagic stroke |
| pipamazine | phenothiazine | antiemetic | 1969 | withdrawn | safety | hepatotoxicity |
| prenylamine | calcium channel blocker | angina pectoris | 1988 | withdrawn | safety | QT prolongation, cardiac arrhythmias |
| rapacuronium | AChE inhibitor | muscle relaxant, anesthesia adjunct | 2001 | withdrawn | safety | fatal bronchospasm |
| remoxipride | D_2_R-mGPCR antagonist | antipsychotic | 1993 | withdrawn | safety | aplastic anemia |
| rimonabant | CB_1_R inverse agonist | anorectic | 2008 | withdrawn | safety | serious psychiatric adverse effects (depression, mood alterations, suicidal ideation), nausea, upper respiratory tract infections |
| sparfloxacin | fluoroquinolone/ DNA gyrase inhibitor | antibacterial drug | 2001 | withdrawn | safety | QT prolongation, phototoxicity |
| sparteine | sodium channel inhibitor | cardiac arrhythmia | 1979 | withdrawn | safety | unpredictable effects, tetanic uterine contractions, obstetrical complications |
| sulfadimethoxine | sulfonamide, dihydropteroate synthase inhibitor | antibacterial drug | 1966 | withdrawn | safety | Stephens-Johnson-syndrome, fatalities |
| suloctidil | calcium antagonist | thrombosis | 1985 | withdrawn | safety | hepatotoxicity, hepatitis |
| ***Drug Name (synonym)*** | ***Drug class*** | ***Formerly FDA-approved indication*** | ***Year of removal*** | ***Status*** | ***Removal category*** | ***Reason of market removal [1, 3-5, 9, 19, 21]*** |
| terfenadine | H_1_R antagonist | allergic rhinitis, allergic skin disorders | 1998 | withdrawn | safety | cardiovascular adverse effects (prolonged QT interval, ventricular tachycardia), drug interactions (CYP3A4) |
| thenalidine | H_1_R antagonist | pruritus | 1963 | withdrawn | safety | neutropenia |
| tribromsalan | halogenated salicylanilides | antimicrobial | 1975 | withdrawn | safety | photosensitivity, serious skin disorders |
| triparanol | cholesterol synthesis inhibitor | hypercholesterinemia | 1962 | withdrawn | safety | nausea, vomiting, ocular toxicity (vision loss, irreversible cataracts), dermatological toxicity (alopecia, ichthyosis) |
| troglitazone | PPAR-α agonist | diabetes type 2 | 2000 | withdrawn | safety | hepatotoxicity |
| trovafloxacin | fluoroquinolone, DNA gyrase inhibitor | antibacterial drug, STI (gonorrhea, chlamydia) | 2006 | withdrawn | safety | hepatotoxicity |
| tryptophan | amino acid | depression | 1989 | withdrawn | safety | musculoskeletal and hematological toxicity, eosinophilia-myalgia syndrome |
| zimelidine | SSRI | depression | 1983 | withdrawn | safety | peripheral neuropathy, Guillain-Barré syndrome, hypersensitivity reaction (exanthema, arthralgias, eosinophilia), psychiatric adverse effects (suicidal ideation) |
| fenfluramine | 5-HT_x_R agonist | anorectic, Dravet syndrome | 1997, 2024 | withdrawn, reapproved | safety | heart valve disease, pulmonary hypertension, cardiac fibrosis; reintroduced for different indication (Dravet syndrome) |

***Supplemental Table 2:*** *Overview about drug discontinuations from the US market. Given is the drugs name, its removal year (unknown dates stated as “?”), its status, its primary removal cause, its indication, and drug class. Drug removals that were initially discontinued by the manufacturer but later withdrawn by the FDA are listed under withdrawn drugs. Drugs are sorted alphabetically.*

| **Drug Name (synonym)** | **Drug class** | **Formerly FDA-approved indication** | **Year of removal** | **Status** | **Removal category** | **Reason of market removal [1, 3-5, 9, 19, 21]** |
| --- | --- | --- | --- | --- | --- | --- |
| alatrofloxacin | fluoroquinolone, DNA gyrase inhibitor | antibacterial drug | 2006 | discontinued | safety | hepatotoxicity (liver injury, death) |
| aminopterin | folic acid derivative | antineoplastic drug (pediatric leukemia) | 1960 | discontinued | manufacturer choice | replacement in usage by methotrexate with lower toxicity; manufacturing difficulties |
| antazoline | H_1_R antagonist | allergic symptoms | 1994 | discontinued | manufacturer choice | business decision (not specified) |
| ardeparin | low molecular weight heparin | prevention of deep vein thrombosis | 1997 | discontinued | manufacturer choice | business decision (not because of safety/efficacy consideration) |
| astemizole | H_1_R antagonist | allergic symptoms | 1999 | discontinued | safety | cardiac adverse effects (fatal arrhythmia) |
| azatadine | H_1_R antagonist | allergic symptoms | 1982 | discontinued | manufacturer choice | business decision (not specified) |
| benoxaprofen | COX inhibitor | arthritis | 1982 | discontinued | safety | liver and kidney failure, gastrointestinal bleeding, fatal cholestatic jaundice |
| bentiromide | serine protease ligand | diagnostic agent (pancreatic exocrine insufficiency) | 1996 | discontinued | manufacturer choice | business decision, declining use because of frequent adverse effects (headache, gastrointestinal disturbances) |
| ***Drug Name (synonym)*** | ***Drug class*** | ***Formerly FDA-approved indication*** | ***Year of removal*** | ***Status*** | ***Removal category*** | ***Reason of market removal [1, 3-5, 9, 19, 21]*** |
| bepridil | calcium channel blocker | hypertension, angina, sustained atrial fibrillation and tachyarrhythmia | 2003 | discontinued | safety | ventricular arrhythmia (torsade de pointes) |
| carbenicillin | beta-lactam; penicillin-binding protein inhibitor | antibacterial drug, urinary tract infections | 2008 | discontinued | manufacturer choice | business decision (not specified) |
| carprofen | COX inhibitor | analgesic, antipyretic, anti-inflammatory | 1995 | discontinued | manufacturer choice | commercial grounds |
| cefamandole nafate | beta-lactam; penicillin-binding protein inhibitor | antibacterial drug | 2009 | discontinued | manufacturer choice | business decision (not specified) |
| cephapirin | beta-lactam; penicillin-binding protein inhibitor | antibacterial drug | 1988 | discontinued | manufacturer choice | business decision (not specified) |
| cerivastatin | HMG-CoA-inhibitor | hypercholesterinemia | 2001 | discontinued | safety | rhabdomyolysis |
| ceruletide | cholecystokinin analogon | paralytic ileus, diagnostic agent in pancreatic malfunction | 2006 | discontinued | manufacturer choice | business decision (not specified) |
| chlormezanone | GABA agonist | anxiety, muscle spasms | 1996 | discontinued | safety | serious cutaneous reactions (SJS) |
| chlorphenesin | muscle relaxant (centrally acting) | muscle pain, muscle spasms | 2014 | discontinued | manufacturer choice | business decision, availability of safer spasmolytics such as benzodiazepines |
| cianidanol | antioxidant flavonoid | immune stimulator, liver disease | 1985 | discontinued | safety | hemolytic anemia |
| ***Drug Name (synonym)*** | ***Drug class*** | ***Formerly FDA-approved indication*** | ***Year of removal*** | ***Status*** | ***Removal category*** | ***Reason of market removal [1, 3-5, 9, 19, 21]*** |
| cinoxacin | fluoroquinolone, DNA gyrase inhibitor | antibacterial drug | 1999 | discontinued | manufacturer choice | business decision, decreasing use because of availability of more favorable and efficacious second-generation fluoroquinolones |
| clofibrate | PPAR-α agonist | hypercholesterinemia, hyperleptinemia | 2002 | discontinued | manufacturer choice | more adverse effects than comparable therapeutic options; availability of fibrates |
| dapiprazole | α-AR antagonist | drug induced mydriasis | 2013 | discontinued | manufacturer choice | slow effect |
| dicumarol | VKA | oral anticoagulant | 1955 | discontinued | manufacturer choice | replaced by warfarin |
| diphemanil | M_x_R antagonist | peptic ulcer, gastric hyperacidity, hypermotility in gastritis and pylorospasm, hyperhidrosis | 1994 | discontinued | manufacturer choice | business decision (not specified) |
| diphenidol | M_x_R antagonist | vomiting and vertigo in Morbus Meniere and labyrinthopathies | 2009 | discontinued | manufacturer choice | business decision (not specified) |
| dydrogesterone | synthetic progesterone | menstrual cycle regulation, infertility treatment | 1993 | discontinued | manufacturer choice | commercial ground (no safety or efficacy reasons) |
| encainide | sodium channel inhibitor | arrhythmia | 1991 | discontinued | safety | ventricular arrhythmia, voluntarily withdrawn |
| enoxacin | fluoroquinolone, DNA gyrase inhibitor | antibacterial drug | 1992 | discontinued | safety | seizures, toxicity |
| ***Drug Name (synonym)*** | ***Drug class*** | ***Formerly FDA-approved indication*** | ***Year of removal*** | ***Status*** | ***Removal category*** | ***Reason of market removal [1, 3-5, 9, 19, 21]*** |
| ethchlorvynol | GABA modulator | insomnia, sedation | 1999 | discontinued | manufacturer choice | superseded by newer drugs; unfavorable risk-benefit, withdrawal symptoms |
| etretinate | retinoid | psoriasis | 1998 | discontinued | safety | risk for birth defects |
| gallamine | AChE inhibitor | muscle-relaxant surgery | 2001 | discontinued | manufacturer choice | business decisions, replaced by newer muscle-relaxants with fewer adverse effects |
| grepafloxacin | fluoroquinolone, DNA gyrase inhibitor | antibacterial drug | 2003 | discontinued | safety | cardiac repolarization, QT interval prolongation |
| hexocyclium | M_x_R antagonist | peptic ulcer, diarrhea, cramps | 2006 | discontinued | manufacturer choice | replaced by other treatment options like PPI and peripheral MOR agonists |
| hydrocortamate | GCR agonist | anti-inflammatory, inflammatory dermatoses | 2001 | discontinued | manufacturer choice | business decision, possibly less favorable safety-efficacy ratio than other GCR agonists (not specified) |
| iodamide | radiographic contrast medium | diagnostic drug | 1987 | discontinued | manufacturer choice | business decision (not specified) |
| iopanoic acid | iodinated ionic monomeric contrast medium | diagnostic drug, cholecystography | ? | discontinued | manufacturer choice | business decision (not specified) |
| levacetyl-methadol | MOR agonist | opioid dependence | 2003 | discontinued | manufacturer choice | business decision, inferior because of QT interval prolongation |
| levallorphan | MOR agonist | reversal of respiratory depression in surgical anesthesia (partial MOR agonist); narcotic overdose | 2001 | discontinued | manufacturer choice | business decision, psychiatric adverse reactions (hallucinations, dissociation, dysphoria, anxiety, confusion, dizziness, disorientation, derealization, feelings of drunkenness, bizarre, unusual or disturbing dreams); replaced by newer and more favorable drugs like naloxone |
| ***Drug Name (synonym)*** | ***Drug class*** | ***Formerly FDA-approved indication*** | ***Year of removal*** | ***Status*** | ***Removal category*** | ***Reason of market removal [1, 3-5, 9, 19, 21]*** |
| lomefloxacin | fluoroquinolone, DNA gyrase inhibitor | antibacterial drug, chronic bronchitis, urinary tract infections | 2009 | discontinued | manufacturer choice | business decision (not specified) |
| loracarbef | beta-lactam; penicillin-binding protein inhibitor | antibacterial drug | 2006 | discontinued | manufacturer choice | business decision (not specified) |
| masoprocol | 5-lipoxygenase inhibitor | actinic keratoses | 1996 | discontinued | manufacturer choice | business decision, allergic reactions |
| mazindol | MAO inhibitor | obesity, Duchenne muscular dystrophy | 1999 | discontinued | manufacturer choice | business decision, low sales, not because safety |
| mephentermine | α-AR agonist | hypotension | 2011 | discontinued | manufacturer choice | business decision, potentially replaced by other alternatives, risk of dependence |
| mesoridazine | D_2_R/H-HT-2 antagonist | schizophrenia, behavioral problems in mental deficiency, chronic brain syndrome, alcoholism, psychoneurotic symptoms | 2008 | discontinued | manufacturer choice | business decision, only second choice because cardiac adverse effects (QT prolongation, arrythmia, torsade de pointes, sudden death) but not discontinued because of efficacy or safety |
| methapyrilene | H_1_R antagonist | insomnia | 1979 | discontinued | safety | potent carcinogen (liver cancer in rats) |
| metharbital | GABA-A agonist, barbiturate | anticonvulsant | 1990 | discontinued | manufacturer choice | business decision, probably because emerging treatment options and obsoleteness of barbiturates for epilepsy |
| ***Drug Name (synonym)*** | ***Drug class*** | ***Formerly FDA-approved indication*** | ***Year of removal*** | ***Status*** | ***Removal category*** | ***Reason of market removal [1, 3-5, 9, 19, 21]*** |
| methicillin | beta-lactam; penicillin-binding protein inhibitor | antibacterial drug | 2010 | discontinued | manufacturer choice | less active than other penicillin, only parenteral administration, higher rate of adverse effects, e.g. interstitial nephritis |
| metipranolol | β-AR antagonist | arrythmia, hypertension, glaucoma | 2001 | discontinued | manufacturer choice | business decision, patent expiration |
| metocurine iodide | ACh antagonist | muscle relaxant (anesthesia, convulsant) | 2001 | discontinued | manufacturer choice | business decision, possibly because of availability of more effective and favorable treatment options |
| mibefradil | calcium channel blocker | hypertension, stable chronic angina | 1998 | discontinued | safety | new information about potentially harmful interactions with other drugs |
| mivacurium | ACh antagonist | muscle relaxant (anesthesia, mechanical ventilation) | 2021 | discontinued | manufacturer choice | business decision (not specified) |
| moricizine | sodium channel inhibitor | ventricular arrhythmia | 2007 | discontinued | manufacturer choice | business decision (not specified) |
| nalidixic acid | fluoroquinolone, DNA gyrase inhibitor | antibacterial drug, urinary tract infections | 1992 | discontinued | manufacturer choice | business decision, replacement by newer fluoroquinolones |
| nialamide | MAO inhibitor | depression | 1963 | discontinued | manufacturer choice | business decision, hepatotoxicity, food interactions (tyrosine) |
| oxphenbutazone | COX inhibitor | rheumatic disorders (ankylosing spondylitis, osteoarthritis, rheumatoid arthritis) | 1984 | discontinued | safety | bone marrow suppression, Stephens Johnson syndrome |
| pargyline | MAO-B inhibitor | hypertension | 2007 | discontinued | manufacturer choice | limited use due to adverse effects (orthostatic hypotension) and interactions (serotonergic syndrome, hypertensive crisis) |
| ***Drug Name (synonym)*** | ***Drug class*** | ***Formerly FDA-approved indication*** | ***Year of removal*** | ***Status*** | ***Removal category*** | ***Reason of market removal [1, 3-5, 9, 19, 21]*** |
| pipobroman | DNA alkylation | cancer treatment, polycythemia vera, essential thrombocythemia | 2015 | discontinued | manufacturer choice | business decision (not specified) |
| pirprofen | COX inhibitor | rheumatic disorders (osteoarthritis, rheumatoid arthritis), analgesic | 1990 | discontinued | safety | fatal hepatotoxicity |
| plicamycin | DNA antagonist | cancer treatment, hypercalcemia and hypercalciuria during cytotoxic treatment, Paget's disease | 2000 | discontinued | manufacturer choice | business decision (not specified) |
| potassium arsenite | inorganic trivalent arsenic | acute promyelocytic leukemia, several historical uses | 1980 | discontinued | safety | carcinogenic, increased risk of cardiovascular abnormalities, diabetes mellitus, neurotoxicity and nephrotoxicity |
| probucol | ABCA_1_ inhibitor | hypercholesterinemia | 1995 | discontinued | safety | cardiac adverse effects (prolonged QT interval, fatal arrhythmia) |
| quinethazone | NCC inhibitor | thiazide diuretic, hypertension | 1989 | discontinued | manufacturer choice | business decision, possibly replaced by newer NCC inhibitors |
| ritodrine | β-AR agonist | stop of premature labor | 1998 | discontinued | safety | severe maternal cardiopulmonary adverse effects |
| rofecoxib | COX inhibitor | arthritis, acute pain in adults, painful menstrual cycles | 2004 | discontinued | safety | heart attack, stroke |
| sibutramine | sodium-dependent Dopamine-/Serotonin-/ Noradrenaline- transporter inhibitor | anorectic | 2010 | discontinued | safety | heart attack, stroke |
| ***Drug Name (synonym)*** | ***Drug class*** | ***Formerly FDA-approved indication*** | ***Year of removal*** | ***Status*** | ***Removal category*** | ***Reason of market removal [1, 3-5, 9, 19, 21]*** |
| sitaxentan | endothelin receptor antagonist | pulmonary arterial hypertension | 2010 | discontinued | safety | life-threatening idiosyncratic risk of liver injury |
| sulfacytine | sulfonamide, dihydropteroate synthase inhibitor | antibacterial drug, uncomplicated urinary tract infections | 2006 | discontinued | manufacturer choice | business decision, probably replaced by other antibacterial drugs |
| sulfamethazine | sulfonamide, dihydropteroate synthase inhibitor | antibacterial drug | ? | discontinued | manufacturer choice | business decision, probably replaced by other antibacterial drugs (unfavorable risk-benefit ratio) |
| sulfapyridine | sulfonamide, dihydropteroate synthase inhibitor | antibacterial drug, dermatitis herpetiformis | 1990 | discontinued | manufacturer choice | business decision (not specified), probably unfavorable risk-benefit ratio and availability of superior treatment options |
| sulfinpyrazone | hURAT_1_/ hOAT_4_/ COX/ CFTR inhibitor | uricosuric, chronic or intermittent gouty arthritis | 2009 | discontinued | manufacturer choice | business decision (not specified) |
| suprofen | COX inhibitor | inhibition of intraoperative miosis | 1988 | discontinued | safety | flank pain, decreased kidney function |
| tacrine | AChE inhibitor | Alzheimer dementia | 2013 | discontinued | manufacturer choice | business decision, liver toxicity and requirement for monitoring liver function, low prescriptions and introduction of more favorable AChE inhibitors |
| tegaserod | 5-HT_4_ agonist | short-term treatment of women with irritable bowel syndrome (IBS) with constipation | 2007, 2019 | discontinued | safety | heart attack, stroke, unstable angina pectoris; remarketed under restricted usage conditions |
| ***Drug Name (synonym)*** | ***Drug class*** | ***Formerly FDA-approved indication*** | ***Year of removal*** | ***Status*** | ***Removal category*** | ***Reason of market removal [1, 3-5, 9, 19, 21]*** |
| telaprevir | NS3/4A protease inhibitor | hepatitis C (genotype 1, chronic, adults) | 2015 | discontinued | manufacturer choice | business decision, availability of oral direct acting drugs with superior efficacy and better tolerance, severe cutaneous reactions (DRESS, SJS), hepatic decompensation |
| thiabendazole | helminth specific enzyme fumarate reductase inhibitor | parasitic infections | 1995 | discontinued | manufacturer choice | business decisions, replaced by better tolerated anthelmintic drugs |
| thiethylperazine | D_x_R antagonist | nausea and vomiting in radiotherapy/chemotherapy/postoperative care | 2009 | discontinued | manufacturer choice | business decision (not specified) |
| thiopental | GABA-A agonist, barbiturate | induction of anesthesia, adjunct in convulsion therapy, reduction of intracranial pressure | 2011 | discontinued | manufacturer choice | business decision, political decision of manufacturer because widely used in execution of death in US prisoners |
| tocainide | sodium channel inhibitor | ventricular arrhythmia | 2003 | discontinued | safety | hematological adverse effects, bone marrow suppression |
| tolazoline | α-AR antagonist | persistent pulmonary hypertension in newborn | 2002 | discontinued | manufacturer choice | business decision (not specified) |
| tridihexethyl | M_x_R antagonist | peptic ulcer disease | 2012 | discontinued | manufacturer choice | business decision (not specified), probably because of availability of PPI as treatment option |
| trilostane | 3 β-hydroxysteroid dehydrogenase inhibitor | Cushing's syndrome | 1994 | discontinued | manufacturer choice | inconsistencies in efficacy and concerns over safety profiles (not specified) |
| ***Drug Name (synonym)*** | ***Drug class*** | ***Formerly FDA-approved indication*** | ***Year of removal*** | ***Status*** | ***Removal category*** | ***Reason of market removal [1, 3-5, 9, 19, 21]*** |
| trioxsalen | DNA alkylation | vitiligo | 2002 | discontinued | manufacturer choice | business decision (not specified), probably because of more effective and favorable newer drugs |
| tyloxapol | lipoprotein lipase inhibitor | surfactant | 2003 | discontinued | manufacturer choice | business decision (not specified) |
| valdecoxib | COX inhibitor | arthritis, acute pain in adults, painful menstrual cycles | 2005 | discontinued | safety | heart attack, stroke |
| vidarabine | HHV-1 inhibitor, thymidine kinase inducer | viral infections | 2006 | discontinued | manufacturer choice | business decision, commercial reasons |
| zalcitabine | NRTI inhibitor | HIV | 2006 | discontinued | manufacturer choice | business decision, neurotoxicity (axonal degeneration, peripheral neuropathy), replaced by other more effective treatment options with less adverse effects |
| zomepirac | COX inhibitor | analgesic | 1983 | discontinued | safety | anaphylactic reactions, hypersensitivity reactions |
| alosetron | 5-HT_3_ antagonist | treatment of women with severe irritable bowel syndrome (IBS) with diarrhea | 2000, 2002 | discontinued, reapproved | safety | ischemic colitis, perforated and ruptured bowel; remarketed under restricted usage conditions |
| doxylamine | H_1_R antagonist, ACh antagonist | insomnia, allergic symptoms, nausea and vomiting in pregnancy | 1983, 2013 | discontinued, reapproved | manufacturer choice | consequence of lawsuits about cause of fetal malformation (later disproved) |

***Supplemental Table 3:*** *Characterization of drugs. Given are the status and main reason for removal, and the drugs allocation in usage fields, drug mechanisms, and removal causes. The following market removal reason categories were assigned: (1) business decision, commercial reasons; (2) availability of better treatment options (more efficient or safe), obsoleteness; (3) hepatotoxicity and hepatic adverse effects; (4) nephrotoxicity and renal adverse effects; (5) hematologic toxicity and hematologic adverse reactions; (6) immunologic and hypersensitivity reactions; (7) dermatologic toxicity and cutaneous adverse reactions; (8) ototoxicity; (9) carcinogenicity and induction of malignancies; (10) drug or food interactions; (11) metabolic and endocrine adverse effects, and laboratory abnormalities; (12) tolerance, dependence, misuse, and withdrawal symptoms; (13) gastrointestinal toxicity and gastrointestinal adverse effects; (14) ophthalmic toxicity and visual disturbances; (15) psychiatric and behavioral adverse effects; (16) neurotoxicity and neurological adverse effects; (17) cardiotoxicity and cardiac adverse effects; (18) pulmonary toxicity and respiratory adverse effects; (19) developmental, reproductive, and embryo-fetal toxicity; (20) increased mortality or fatal outcomes; (21) exacerbation or worsening of underlying disease; (22) political decisions of the manufacturer; (23) musculoskeletal toxicity; (24) production difficulties by the manufacturer. Drugs are sorted by usage fields.*

| **Drug Name (synonym)** | **Withdrawn vs. discontinued** | **Removal category** | **Usage field** | **Drug mechanism category** | **Removal cause category** |
| --- | --- | --- | --- | --- | --- |
| aminopyrine (aminophenazone) | withdrawn | safety | analgesic and immunomodulating drugs | miscellaneous | 5, 6 |
| antazoline | discontinued | manufacturer choice | analgesic and immunomodulating drugs | receptor agonist/ antagonist | 1 |
| astemizole | discontinued | safety | analgesic and immunomodulating drugs | receptor agonist/ antagonist | 17 |
| azatadine | discontinued | manufacturer choice | analgesic and immunomodulating drugs | receptor agonist/ antagonist | 1 |
| benoxaprofen | discontinued | safety | analgesic and immunomodulating drugs | enzyme inhibitor | 3, 4, 13 |
| butamben | withdrawn | safety | analgesic and immunomodulating drugs | ion channel modulator | 7, 16 |
| carprofen | discontinued | manufacturer choice | analgesic and immunomodulating drugs | enzyme inhibitor | 1 |
| cianidanol | discontinued | safety | analgesic and immunomodulating drugs | miscellaneous | 5 |
| dimethylamylamine | withdrawn | safety | analgesic and immunomodulating drugs | receptor agonist/ antagonist | 15, 16, 17 |
| dipyrone (metamizole) | withdrawn | safety | analgesic and immunomodulating drugs | miscellaneous | 5, 6 |
| doxylamine | discontinued, reapproved | manufacturer choice | analgesic and immunomodulating drugs | receptor agonist/ antagonist | 1 |
| ethoheptazine | withdrawn | manufacturer choice | analgesic and immunomodulating drugs | receptor agonist/ antagonist | 1, 2 |
| fenclozic acid | withdrawn | safety | analgesic and immunomodulating drugs | enzyme inhibitor | 3 |
| glafenine | withdrawn | safety | analgesic and immunomodulating drugs | enzyme inhibitor | 4, 6 |
| hydrocortamate | discontinued | manufacturer choice | analgesic and immunomodulating drugs | receptor agonist/ antagonist | 1, 2 |
| ***Drug Name (synonym)*** | ***Withdrawn vs. discontinued*** | ***Removal category*** | ***Usage field*** | ***Drug mechanism category*** | ***Removal cause category*** |
| indoprofen | withdrawn | safety | analgesic and immunomodulating drugs | enzyme inhibitor | 13 |
| methopholine | withdrawn | safety | analgesic and immunomodulating drugs | receptor agonist/ antagonist | 14 |
| oxphenbutazone | discontinued | safety | analgesic and immunomodulating drugs | enzyme inhibitor | 5, 6, 7 |
| parecoxib | withdrawn | safety | analgesic and immunomodulating drugs | enzyme inhibitor | 2, 6, 7, 17 |
| phenacetin | withdrawn | safety | analgesic and immunomodulating drugs | enzyme inhibitor | 5, 9 |
| pirprofen | discontinued | safety | analgesic and immunomodulating drugs | enzyme inhibitor | 3 |
| rofecoxib | discontinued | safety | analgesic and immunomodulating drugs | enzyme inhibitor | 17 |
| sulfinpyrazone | discontinued | manufacturer choice | analgesic and immunomodulating drugs | enzyme inhibitor | 1 |
| suprofen | discontinued | safety | analgesic and immunomodulating drugs | enzyme inhibitor | 4 |
| terfenadine | withdrawn | safety | analgesic and immunomodulating drugs | receptor agonist/ antagonist | 10, 17 |
| thenalidine | withdrawn | safety | analgesic and immunomodulating drugs | receptor agonist/ antagonist | 5, 6 |
| valdecoxib | discontinued | safety | analgesic and immunomodulating drugs | enzyme inhibitor | 17 |
| zomepirac | discontinued | safety | analgesic and immunomodulating drugs | enzyme inhibitor | 6 |
| alatrofloxacin | discontinued | safety | anti-infective drugss | enzyme inhibitor | 3 |
| bithionol | withdrawn | safety | anti-infective drugss | enzyme inhibitor | 7 |
| carbenicillin | discontinued | manufacturer choice | anti-infective drugss | enzyme inhibitor | 1 |
| cefamandole nafate | discontinued | manufacturer choice | anti-infective drugss | enzyme inhibitor | 1 |
| cephapirin | discontinued | manufacturer choice | anti-infective drugss | enzyme inhibitor | 1 |
| chloramphenicol | withdrawn | safety | anti-infective drugss | miscellaneous | 5 |
| ***Drug Name (synonym)*** | ***Withdrawn vs. discontinued*** | ***Removal category*** | ***Usage field*** | ***Drug mechanism category*** | ***Removal cause category*** |
| cinoxacin | discontinued | manufacturer choice | anti-infective drugs | enzyme inhibitor | 1, 2 |
| diamthazole | withdrawn | safety | anti-infective drugs | miscellaneous | 15, 16 |
| dibromsalan | withdrawn | safety | anti-infective drugs | miscellaneous | 7 |
| dihydrostreptomycin | withdrawn | safety | anti-infective drugs | miscellaneous | 2, 8 |
| dithiazanine iodide | withdrawn | safety | anti-infective drugs | miscellaneous | 11 |
| enoxacin | discontinued | safety | anti-infective drugs | enzyme inhibitor | 16 |
| grepafloxacin | discontinued | safety | anti-infective drugs | enzyme inhibitor | 17 |
| levamisole | withdrawn | safety | anti-infective drugs | receptor agonist/ antagonist | 5, 6 |
| lomefloxacin | discontinued | manufacturer choice | anti-infective drugs | enzyme inhibitor | 1 |
| loracarbef | discontinued | manufacturer choice | anti-infective drugs | enzyme inhibitor | 1 |
| metabromsalan | withdrawn | safety | anti-infective drugs | miscellaneous | 7 |
| methicillin | discontinued | manufacturer choice | anti-infective drugs | enzyme inhibitor | 2, 4 |
| nalidixic acid | discontinued | manufacturer choice | anti-infective drugs | enzyme inhibitor | 1, 2 |
| novobiocin | discontinued, withdrawn | manufacturer choice | anti-infective drugs | enzyme inhibitor | 2, 3, 5, 7, 10 |
| sparfloxacin | withdrawn | safety | anti-infective drugs | enzyme inhibitor | 7, 17 |
| sulfacytine | discontinued | manufacturer choice | anti-infective drugs | enzyme inhibitor | 1, 2 |
| sulfadimethoxine | withdrawn | safety | anti-infective drugs | enzyme inhibitor | 6, 7, 20 |
| sulfamethazine | discontinued | manufacturer choice | anti-infective drugs | enzyme inhibitor | 1, 2 |
| sulfapyridine | discontinued | manufacturer choice | anti-infective drugs | enzyme inhibitor | 1, 2 |
| sulfathiazole | discontinued/ withdrawn | safety | anti-infective drugs | enzyme inhibitor | 3, 4, 5, 6, 7 |
| telaprevir | discontinued | manufacturer choice | anti-infective drugs | enzyme inhibitor | 1, 2, 3, 6, 7 |
| ***Drug Name (synonym)*** | ***Withdrawn vs. discontinued*** | ***Removal category*** | ***Usage field*** | ***Drug mechanism category*** | ***Removal cause category*** |
| temafloxacin | discontinued, withdrawn | safety | anti-infective drugs | enzyme inhibitor | 3, 4, 5, 6, 11 |
| thiabendazole | discontinued | manufacturer choice | anti-infective drugs | miscellaneous | 1, 2 |
| tribromsalan | withdrawn | safety | anti-infective drugs | miscellaneous | 7 |
| trovafloxacin | withdrawn | safety | anti-infective drugs | enzyme inhibitor | 3 |
| vidarabine | discontinued | manufacturer choice | anti-infective drugs | miscellaneous | 1 |
| zalcitabine | discontinued | manufacturer choice | anti-infective drugs | enzyme inhibitor | 2, 16 |
| adenosine phosphate | withdrawn | safety | cardiovascular and renal drugs | enzyme inhibitor | 17 |
| aprotinin | withdrawn | safety | cardiovascular and renal drugs | enzyme inhibitor | 20 |
| ardeparin | discontinued | manufacturer choice | cardiovascular and renal drugs | enzyme inhibitor | 1, 2 |
| bepridil | discontinued | safety | cardiovascular and renal drugs | ion channel modulator | 17 |
| dapiprazole | discontinued | manufacturer choice | cardiovascular and renal drugs | receptor agonist/ antagonist | 1, 2 |
| dicumarol | discontinued | manufacturer choice | cardiovascular and renal drugs | enzyme inhibitor | 1, 2 |
| drotrecogin alfa | withdrawn | efficacy | cardiovascular and renal drugs | enzyme inhibitor | 2 |
| encainide | discontinued | safety | cardiovascular and renal drugs | ion channel modulator | 17 |
| ethyl biscoumacetate | withdrawn | safety | cardiovascular and renal drugs | enzyme inhibitor | 5 |
| flosequinan | withdrawn | safety, efficacy | cardiovascular and renal drugs | enzyme inhibitor | 20, 21 |
| mephentermine | discontinued | manufacturer choice | cardiovascular and renal drugs | receptor agonist/ antagonist | 1, 2, 12 |
| metipranolol | discontinued | manufacturer choice | cardiovascular and renal drugs | receptor agonist/ antagonist | 1 |
| mibefradil | discontinued | safety | cardiovascular and renal drugs | ion channel modulator | 10 |
| ***Drug Name (synonym)*** | ***Withdrawn vs. discontinued*** | ***Removal category*** | ***Usage field*** | ***Drug mechanism category*** | ***Removal cause category*** |
| moricizine | discontinued | manufacturer choice | cardiovascular and renal drugs | ion channel modulator | 1 |
| muzolimine | withdrawn | safety | cardiovascular and renal drugs | ion channel modulator | 16 |
| pargyline | discontinued | manufacturer choice | cardiovascular and renal drugs | enzyme inhibitor | 16, 17 |
| phenylpropanolamine | withdrawn | safety | cardiovascular and renal drugs | receptor agonist/ antagonist | 17 |
| prenylamine | withdrawn | safety | cardiovascular and renal drugs | ion channel modulator | 17 |
| quinethazone | discontinued | manufacturer choice | cardiovascular and renal drugs | ion channel modulator | 1, 2 |
| ritodrine | discontinued | safety | cardiovascular and renal drugs | receptor agonist/ antagonist | 17, 18 |
| sitaxentan | discontinued | safety | cardiovascular and renal drugs | receptor agonist/ antagonist | 3 |
| sparteine | withdrawn | safety | cardiovascular and renal drugs | ion channel modulator | 13, 18 |
| suloctidil | withdrawn | safety | cardiovascular and renal drugs | ion channel modulator | 3 |
| ticrynafen (tienilic acid) | discontinued, withdrawn | safety | cardiovascular and renal drugs | ion channel modulator | 3 |
| tocainide | discontinued | safety | cardiovascular and renal drugs | ion channel modulator | 5 |
| tolazoline | discontinued | manufacturer choice | cardiovascular and renal drugs | receptor agonist/ antagonist | 1 |
| alphacetylmethadol | withdrawn | safety | central nervous system | receptor agonist/ antagonist | 17 |
| alpha-ethyltryptamine (etryptamine) | withdrawn | safety | central nervous system | enzyme inhibitor | 5, 6, 12 |
| althesin | withdrawn | safety | central nervous system | miscellaneous | 6 |
| ***Drug Name (synonym)*** | ***Withdrawn vs. discontinued*** | ***Removal category*** | ***Usage field*** | ***Drug mechanism category*** | ***Removal cause category*** |
| benactyzine | withdrawn | safety | central nervous system | receptor agonist/ antagonist | 15, 16 |
| chlormezanone | discontinued | safety | central nervous system | miscellaneous | 6, 7 |
| chloroform | withdrawn | safety | central nervous system | miscellaneous | 9 |
| chlorphenesin | discontinued | manufacturer choice | central nervous system | miscellaneous | 1, 2 |
| dexfenfluramine | withdrawn | safety | central nervous system | receptor agonist/ antagonist | 17 |
| dextropropoxyphene | withdrawn | safety | central nervous system | receptor agonist/ antagonist | 17 |
| diphenidol | discontinued | manufacturer choice | central nervous system | receptor agonist/ antagonist | 1 |
| ethchlorvynol | discontinued | manufacturer choice | central nervous system | miscellaneous | 1, 2, 12 |
| fenfluramine | withdrawn, reapproved | safety | central nervous system | receptor agonist/ antagonist | 17 |
| gallamine | discontinued | manufacturer choice | central nervous system | receptor agonist/ antagonist | 1, 2 |
| iproniazid | withdrawn | safety | central nervous system | enzyme inhibitor | 3 |
| levacetylmethadol | discontinued | manufacturer choice | central nervous system | receptor agonist/ antagonist | 1, 17 |
| levallorphan | discontinued | manufacturer choice | central nervous system | receptor agonist/ antagonist | 1, 2, 15 |
| mazindol | discontinued | manufacturer choice | central nervous system | enzyme inhibitor | 1 |
| ***Drug Name (synonym)*** | ***Withdrawn vs. discontinued*** | ***Removal category*** | ***Usage field*** | ***Drug mechanism category*** | ***Removal cause category*** |
| mesoridazine | discontinued | manufacturer choice | central nervous system | receptor agonist/ antagonist | 1, 2, 17 |
| methapyrilene | discontinued | safety | central nervous system | receptor agonist/ antagonist | 9 |
| methaqualone | withdrawn | safety | central nervous system | miscellaneous | 12 |
| metharbital | discontinued | manufacturer choice | central nervous system | miscellaneous | 1, 2 |
| methoxyflurane | discontinued, withdrawn | safety | central nervous system | miscellaneous | 3, 4 |
| metocurine iodide | discontinued | manufacturer choice | central nervous system | receptor agonist/ antagonist | 1, 2 |
| mivacurium | discontinued | manufacturer choice | central nervous system | receptor agonist/ antagonist | 1 |
| nialamide | discontinued | manufacturer choice | central nervous system | enzyme inhibitor | 1, 2, 3, 10 |
| nomifensine | discontinued, withdrawn | safety | central nervous system | enzyme inhibitor | 3, 5, 6 |
| oxeladin | withdrawn | safety | central nervous system | miscellaneous | 9 |
| pemoline | discontinued, withdrawn | manufacturer choice | central nervous system | miscellaneous | 1, 3, 5, 15, 16 |
| pentetrazol (pentylenetetrazol) | withdrawn | safety, efficacy | central nervous system | miscellaneous | 2, 16 |
| pergolide | withdrawn | safety | central nervous system | receptor agonist/ antagonist | 17, 21 |
| pipamazine | withdrawn | safety | central nervous system | receptor agonist/ antagonist | 3 |
| ***Drug Name (synonym)*** | ***Withdrawn vs. discontinued*** | ***Removal category*** | ***Usage field*** | ***Drug mechanism category*** | ***Removal cause category*** |
| rapacuronium | withdrawn | safety | central nervous system | receptor agonist/ antagonist | 18 |
| remoxipride | withdrawn | safety | central nervous system | receptor agonist/ antagonist | 5 |
| sibutramine | discontinued | safety | central nervous system | miscellaneous | 17 |
| tacrine | discontinued | manufacturer choice | central nervous system | receptor agonist/ antagonist | 1, 2, 3 |
| thiethylperazine | discontinued | manufacturer choice | central nervous system | receptor agonist/ antagonist | 1 |
| thiopental | discontinued | manufacturer choice | central nervous system | miscellaneous | 1, 22 |
| tryptophan | withdrawn | safety | central nervous system | miscellaneous | 5, 23 |
| zimelidine | withdrawn | safety | central nervous system | miscellaneous | 6, 15, 16 |
| aminopterin | discontinued | manufacturer choice | cytotoxic treatment | miscellaneous | 2, 24 |
| azaribine | withdrawn | safety | cytotoxic treatment | miscellaneous | 5 |
| chlornaphazine | withdrawn | safety | cytotoxic treatment | miscellaneous | 9 |
| etretinate | discontinued | safety | cytotoxic treatment | receptor agonist/ antagonist | 19 |
| masoprocol | discontinued | manufacturer choice | cytotoxic treatment | enzyme inhibitor | 1, 2, 6 |
| mepazine hydrochloride | withdrawn | safety | cytotoxic treatment | enzyme inhibitor | 3, 5, 13, 16, 17 |
| pipobroman | discontinued | manufacturer choice | cytotoxic treatment | miscellaneous | 1 |
| plicamycin | discontinued | manufacturer choice | cytotoxic treatment | miscellaneous | 1 |
| potassium arsenite | discontinued | safety | cytotoxic treatment | miscellaneous | 4, 9, 12, 16, 17 |
| ***Drug Name (synonym)*** | ***Withdrawn vs. discontinued*** | ***Removal category*** | ***Usage field*** | ***Drug mechanism category*** | ***Removal cause category*** |
| trioxsalen | discontinued | manufacturer choice | cytotoxic treatment | miscellaneous | 1, 2 |
| bentiromide | discontinued | manufacturer choice | diagnostic drugs | enzyme inhibitor | 13, 16 |
| bunamiodyl | withdrawn | safety | diagnostic drugs | miscellaneous | 4 |
| ceruletide | discontinued | manufacturer choice | diagnostic drugs | receptor agonist/ antagonist | 1 |
| iodamide | discontinued | manufacturer choice | diagnostic drugs | miscellaneous | 1 |
| iopanoic acid | discontinued | manufacturer choice | diagnostic drugs | miscellaneous | 1 |
| adrenal cortex | withdrawn | efficacy, safety | endocrine system | receptor agonist/ antagonist | 2 |
| chlormadinone | discontinued, withdrawn | safety | endocrine system | receptor agonist/ antagonist | 9 |
| dydrogesterone | discontinued | manufacturer choice | endocrine system | receptor agonist/ antagonist | 1 |
| metandienone (methandrostenolone) | withdrawn | safety | endocrine system | receptor agonist/ antagonist | 12 |
| trilostane | discontinued | manufacturer choice | endocrine system | enzyme inhibitor | 1, 2 |
| alosetron | discontinued, reapproved | safety | metabolism, gastrointestinal and respiratory system | receptor agonist/ antagonist | 13 |
| cerivastatin | discontinued | safety | metabolism, gastrointestinal and respiratory system | enzyme inhibitor | 23 |
| cisapride | withdrawn | safety | metabolism, gastrointestinal and respiratory system | receptor agonist/ antagonist | 17 |
| ***Drug Name (synonym)*** | ***Withdrawn vs. discontinued*** | ***Removal category*** | ***Usage field*** | ***Drug mechanism category*** | ***Removal cause category*** |
| clofibrate | discontinued | manufacturer choice | metabolism, gastrointestinal and respiratory system | receptor agonist/ antagonist | 1, 2 |
| danthron | withdrawn | safety | metabolism, gastrointestinal and respiratory system | miscellaneous | 9 |
| diphemanil | discontinued | manufacturer choice | metabolism, gastrointestinal and respiratory system | receptor agonist/ antagonist | 1 |
| etamivan | withdrawn | safety | metabolism, gastrointestinal and respiratory system | miscellaneous | 16, 18 |
| ethyl nitrite  (sweet spirits of nitre) | withdrawn | safety | metabolism, gastrointestinal and respiratory system | miscellaneous | 5 |
| hexocyclium | discontinued | manufacturer choice | metabolism, gastrointestinal and respiratory system | receptor agonist/ antagonist | 1, 2 |
| nikethamide | withdrawn | safety | metabolism, gastrointestinal and respiratory system | miscellaneous | 12 |
| oxyphenisatin,  oxyphenisation acetate | withdrawn | safety | metabolism, gastrointestinal and respiratory system | miscellaneous | 3 |
| phenformin | withdrawn | safety | metabolism, gastrointestinal and respiratory system | enzyme inhibitor | 11 |
| phenolphthalein | withdrawn | safety | metabolism, gastrointestinal and respiratory system | miscellaneous | 9 |
| probucol | discontinued | safety | metabolism, gastrointestinal and respiratory system | enzyme inhibitor | 17 |
| ***Drug Name (synonym)*** | ***Withdrawn vs. discontinued*** | ***Removal category*** | ***Usage field*** | ***Drug mechanism category*** | ***Removal cause category*** |
| rimonabant | withdrawn | safety | metabolism, gastrointestinal and respiratory system | receptor agonist/ antagonist | 13, 15, 18 |
| tegaserod | discontinued | safety | metabolism, gastrointestinal and respiratory system | receptor agonist/ antagonist | 17 |
| tridihexethyl | discontinued | manufacturer choice | metabolism, gastrointestinal and respiratory system | receptor agonist/ antagonist | 1, 2 |
| triparanol | withdrawn | safety | metabolism, gastrointestinal and respiratory system | enzyme inhibitor | 7, 13, 14 |
| troglitazone | withdrawn | safety | metabolism, gastrointestinal and respiratory system | receptor agonist/ antagonist | 3 |
| tyloxapol | discontinued | manufacturer choice | metabolism, gastrointestinal and respiratory system | miscellaneous | 1 |

***Supplemental Table 4:***  *Comparison of US market removed drugs with the market status of the EU (last updated: March 22 2026). The market status is colored green if the drug is approved and marketed, yellow if approved but discontinued (not marketed), and orange if not approved (not marketed). Both market statuses are compared and colored green if they are consistent (not marketed recently) or discrepant (marketed vs not marketed). Drugs are sorted by the comparison and the US market status.*

| **Drug name (synonym)** | **US market status [2]** | **EU market status [55, 56]** | **comparison US vs. EU** |
| --- | --- | --- | --- |
| alosetron | approved | not approved | discrepancy |
| antazoline | discontinued | marketed, approved (list of nationally authorized medicinal products) | discrepancy |
| cefamandole nafate | discontinued | marketed, approved (list of nationally authorized medicinal products) | discrepancy |
| dapiprazole | discontinued | marketed, approved (list of nationally authorized medicinal products) | discrepancy |
| dydrogesterone | discontinued | marketed, approved (list of nationally authorized medicinal products) | discrepancy |
| mivacurium | discontinued | marketed, approved (list of nationally authorized medicinal products) | discrepancy |
| pipobroman | discontinued | marketed, approved (list of nationally authorized medicinal products) | discrepancy |
| ritodrine | discontinued | marketed, approved (list of nationally authorized medicinal products) | discrepancy |
| sulfathiazole | discontinued | marketed, approved (list of nationally authorized medicinal products) | discrepancy |
| thiethylperazine | discontinued | marketed, approved (list of nationally authorized medicinal products) | discrepancy |
| thiopental | discontinued | marketed, approved (list of nationally authorized medicinal products) | discrepancy |
| aminopyrine (aminophenazone) | withdrawn | approved (list of nationally authorized medicinal products) | discrepancy |
| aprotinin | withdrawn | approved (list of nationally authorized medicinal products) | discrepancy |
| chloramphenicol | withdrawn | approved (list of nationally authorized medicinal products) | discrepancy |
| chlormadinone | withdrawn | approved (list of nationally authorized medicinal products) | discrepancy |
| cisapride | withdrawn | approved (list of nationally authorized medicinal products) | discrepancy |
| dextropropoxyphene | withdrawn | approved (list of nationally authorized medicinal products) | discrepancy |
| iproniazid | withdrawn | approved (list of nationally authorized medicinal products) | discrepancy |
| levamisole | withdrawn | approved (list of nationally authorized medicinal products) | discrepancy |
| methoxyflurane | withdrawn | approved (list of nationally authorized medicinal products) | discrepancy |
| nikethamide | withdrawn | approved (list of nationally authorized medicinal products) | discrepancy |
| ***Drug name (synonym)*** | ***US market status [2]*** | ***EU market status [55, 56]*** | ***comparison US vs. EU*** |
| oxeladin | withdrawn | approved (list of nationally authorized medicinal products) | discrepancy |
| pentetrazol (pentylenetetrazol) | withdrawn | approved (list of nationally authorized medicinal products) | discrepancy |
| phenolphthalein | withdrawn | approved (list of nationally authorized medicinal products) | discrepancy |
| phenylpropanolamine | withdrawn | approved (list of nationally authorized medicinal products) | discrepancy |
| tryptophan | withdrawn | authorized (authorized by EMA as amino acid, not for depression) | discrepancy |
| fenfluramine | approved | approved (authorized by EMA) | consistent |
| doxylamine | approved | marketed, approved (list of nationally authorized medicinal products) | consistent |
| telaprevir | discontinued | discontinued, approval expired | consistent |
| ardeparin | discontinued | not approved | consistent |
| azatadine | discontinued | not approved | consistent |
| bentiromide | discontinued | not approved | consistent |
| bepridil | discontinued | not approved | consistent |
| carbenicillin | discontinued | not approved | consistent |
| carprofen | discontinued | not approved | consistent |
| cephapirin | discontinued | not approved | consistent |
| cerivastatin | discontinued | not approved | consistent |
| ceruletide | discontinued | not approved | consistent |
| chlorphenesin | discontinued | not approved | consistent |
| cinoxacin | discontinued | not approved | consistent |
| clofibrate | discontinued | not approved | consistent |
| dicumarol | discontinued | not approved | consistent |
| diphemanil | discontinued | not approved | consistent |
| diphenidol | discontinued | not approved | consistent |
| enoxacin | discontinued | not approved | consistent |
| ethchlorvynol | discontinued | not approved | consistent |
| etretinate | discontinued | not approved | consistent |
| gallamine | discontinued | not approved | consistent |
| grepafloxacin | discontinued | not approved | consistent |
| ***Drug name (synonym)*** | ***US market status [2]*** | ***EU market status [55, 56]*** | ***comparison US vs. EU*** |
| hexocyclium | discontinued | not approved | consistent |
| hydrocortamate | discontinued | not approved | consistent |
| iodamide | discontinued | not approved | consistent |
| iopanoic acid | discontinued | not approved | consistent |
| levallorphan | discontinued | not approved | consistent |
| lomefloxacin | discontinued | not approved | consistent |
| loracarbef | discontinued | not approved | consistent |
| masoprocol | discontinued | not approved | consistent |
| mazindol | discontinued | not approved | consistent |
| mephentermine | discontinued | not approved | consistent |
| mesoridazine | discontinued | not approved | consistent |
| metharbital | discontinued | not approved | consistent |
| methicillin | discontinued | not approved | consistent |
| metipranolol | discontinued | not approved | consistent |
| metocurine iodide | discontinued | not approved | consistent |
| moricizine | discontinued | not approved | consistent |
| nalidixic acid | discontinued | not approved | consistent |
| oxphenbutazone | discontinued | not approved | consistent |
| pargyline | discontinued | not approved | consistent |
| plicamycin | discontinued | not approved | consistent |
| quinethazone | discontinued | not approved | consistent |
| rofecoxib | discontinued | not approved | consistent |
| sulfacytine | discontinued | not approved | consistent |
| sulfamethazine | discontinued | not approved | consistent |
| sulfapyridine | discontinued | not approved | consistent |
| sulfinpyrazone | discontinued | not approved | consistent |
| suprofen | discontinued | not approved | consistent |
| tacrine | discontinued | not approved | consistent |
| ***Drug name (synonym)*** | ***US market status [2]*** | ***EU market status [55, 56]*** | ***comparison US vs. EU*** |
| thiabendazole | discontinued | not approved | consistent |
| tocainide | discontinued | not approved | consistent |
| tolazoline | discontinued | not approved | consistent |
| tridihexethyl | discontinued | not approved | consistent |
| trioxsalen | discontinued | not approved | consistent |
| tyloxapol | discontinued | not approved | consistent |
| vidarabine | discontinued | not approved | consistent |
| zalcitabine | discontinued | not approved | consistent |
| tegaserod | discontinued | refused | consistent |
| alatrofloxacin | discontinued | withdrawn | consistent |
| levacetylmethadol | discontinued | withdrawn | consistent |
| adenosine phosphate | withdrawn | not approved | consistent |
| adrenal cortex | withdrawn | not approved | consistent |
| alpha-ethyltryptamine (etryptamine) | withdrawn | not approved | consistent |
| althesin | withdrawn | not approved | consistent |
| aminopterin | withdrawn | not approved | consistent |
| astemizole | withdrawn | not approved | consistent |
| azaribine | withdrawn | not approved | consistent |
| benactyzine | withdrawn | not approved | consistent |
| benoxaprofen | withdrawn | not approved | consistent |
| bithionol | withdrawn | not approved | consistent |
| bunamiodyl | withdrawn | not approved | consistent |
| butamben | withdrawn | not approved | consistent |
| chlormezanone | withdrawn | not approved | consistent |
| chlornaphazine | withdrawn | not approved | consistent |
| chloroform | withdrawn | not approved | consistent |
| cianidanol | withdrawn | not approved | consistent |
| danthron | withdrawn | not approved | consistent |
| ***Drug name (synonym)*** | ***US market status [2]*** | ***EU market status [55, 56]*** | ***comparison US vs. EU*** |
| dexfenfluramine | withdrawn | not approved | consistent |
| diamthazole | withdrawn | not approved | consistent |
| dibromsalan | withdrawn | not approved | consistent |
| dihydrostreptomycin | withdrawn | not approved | consistent |
| dimethylamylamine | withdrawn | not approved | consistent |
| dipyrone (metamizole) | withdrawn | not approved | consistent |
| dithiazanine iodide | withdrawn | not approved | consistent |
| encainide | withdrawn | not approved | consistent |
| etamivan | withdrawn | not approved | consistent |
| ethoheptazine | withdrawn | not approved | consistent |
| ethyl biscoumacetate | withdrawn | not approved | consistent |
| ethyl nitrite (sweet spirits of nitre) | withdrawn | not approved | consistent |
| fenclozic acid | withdrawn | not approved | consistent |
| flosequinan | withdrawn | not approved | consistent |
| glafenine | withdrawn | not approved | consistent |
| indoprofen | withdrawn | not approved | consistent |
| mepazine hydrochloride | withdrawn | not approved | consistent |
| metabromsalan | withdrawn | not approved | consistent |
| metandienone (methandrostenolone) | withdrawn | not approved | consistent |
| methapyrilene | withdrawn | not approved | consistent |
| methaqualone | withdrawn | not approved | consistent |
| methopholine | withdrawn | not approved | consistent |
| mibefradil | withdrawn | not approved | consistent |
| muzolimine | withdrawn | not approved | consistent |
| nialamide | withdrawn | not approved | consistent |
| nomifensine | withdrawn | not approved | consistent |
| novobiocin | withdrawn | not approved | consistent |
| oxyphenisatin, oxyphenisation acetate | withdrawn | not approved | consistent |
| ***Drug name (synonym)*** | ***US market status [2]*** | ***EU market status [55, 56]*** | ***comparison US vs. EU*** |
| pemoline | withdrawn | not approved | consistent |
| pergolide | withdrawn | not approved | consistent |
| phenacetin | withdrawn | not approved | consistent |
| phenformin | withdrawn | not approved | consistent |
| pipamazine | withdrawn | not approved | consistent |
| pirprofen | withdrawn | not approved | consistent |
| potassium arsenite | withdrawn | not approved | consistent |
| prenylamine | withdrawn | not approved | consistent |
| probucol | withdrawn | not approved | consistent |
| rapacuronium | withdrawn | not approved | consistent |
| remoxipride | withdrawn | not approved | consistent |
| sibutramine | withdrawn | not approved | consistent |
| sparfloxacin | withdrawn | not approved | consistent |
| sparteine | withdrawn | not approved | consistent |
| sulfadimethoxine | withdrawn | not approved | consistent |
| suloctidil | withdrawn | not approved | consistent |
| temafloxacin | withdrawn | not approved | consistent |
| terfenadine | withdrawn | not approved | consistent |
| thenalidine | withdrawn | not approved | consistent |
| ticrynafen (tienilic acid) | withdrawn | not approved | consistent |
| tribromsalan | withdrawn | not approved | consistent |
| trilostane | withdrawn | not approved | consistent |
| triparanol | withdrawn | not approved | consistent |
| troglitazone | withdrawn | not approved | consistent |
| zimelidine | withdrawn | not approved | consistent |
| zomepirac | withdrawn | not approved | consistent |
| alphacetylmethadol | withdrawn | withdrawn | consistent |
| drotrecogin alfa | withdrawn | withdrawn | consistent |
| ***Drug name (synonym)*** | ***US market status [2]*** | ***EU market status [55, 56]*** | ***comparison US vs. EU*** |
| parecoxib | withdrawn | withdrawn | consistent |
| rimonabant | withdrawn | withdrawn | consistent |
| sitaxentan | withdrawn | withdrawn | consistent |
| trovafloxacin | withdrawn | withdrawn | consistent |
| valdecoxib | withdrawn | withdrawn | consistent |

***Supplemental Table 5:*** *Overview about the publication peak (derived from PubMed, accessed March 15 2026) and its relation to the drug removal. Given is the year of the highest number of publications, the year of drug removal, whether the drug was removed prior, after, similar, or unspecific to the publication peak, and the delay in years between the drug removal vs publication peak. Removed drugs are sorted alphabetically.*

| **Drug Name (synonym)** | **Year of drug removal** | **publication peak** | **publication peak: prior or after removal of US market** | **difference between removal and publication peak (years)** |
| --- | --- | --- | --- | --- |
| adenosine phosphate | 1973 | 2022 | after | 49 |
| adrenal cortex | 1978 | - | - | - |
| alatrofloxacin | 2006 | 1998, 1999, 2000 | before | 6-8 |
| alosetron | 2000 | 2001 | after | 1 |
| alphacetylmethadol | 2003 | 1976 | before | 27 |
| alpha-ethyltryptamine (etryptamine) | 1962 | 1961 | before | 1 |
| althesin | 1984 | 1980 | before | 4 |
| aminopterin | 1960 | 2014 | after | 54 |
| aminopyrine (aminophenazone) | 2000 | 1985 | before | 15 |
| antazoline | 1994 | 1975 | before | 19 |
| aprotinin | 2007 | 1996 | before | 11 |
| ardeparin | 1997 | 1999 | after | 2 |
| astemizole | 1999 | 1993 | before | 6 |
| azaribine | 1977 | 1976 | before | 1 |
| azatadine | 1982 | 1984 | after | 2 |
| benactyzine | ? | 1968 | ? | ? |
| benoxaprofen | 1982 | 1982 | same year | 0 |
| bentiromide | 1996 | 1984 | before | 12 |
| bepridil | 2003 | 2001 | before | 2 |
| bithionol | 1967 | 1975 | after | 8 |
| bunamiodyl | 1963 | 1960 | before | 3 |
| butamben | 1964 | 1991, 1995, 2000, 2024 | after | 27-60 |
| carbenicillin | 2008 | 1974 | before | 34 |
| ***Drug Name (synonym)*** | ***Year of removal*** | ***publication peak*** | ***publication peak: prior or after removal of US market*** | ***difference between removal and publication peak (years)*** |
| carprofen | 1995 | 2015 | after | 20 |
| cefamandole nafate | 2009 | 1978, 1980 | before | 29-31 |
| cephapirin | 1988 | 1980, 1976 | before | 4-8 |
| cerivastatin | 2001 | 2002 | after | 1 |
| ceruletide | 2006 | 1985 | before | 21 |
| chloramphenicol | 2011 | 1992 | before | 19 |
| chlormadinone | 1970, 1972 | 1970 | prior until same year | 0-2 |
| chlormezanone | 1996 | 1960 | before | 36 |
| chlornaphazine | 1964 | 1964 | same year | 0 |
| chloroform | 1976 | 2011 | after | 35 |
| chlorphenesin | 2014 | 1967, 1969, 1977 | before | 37-47 |
| cianidanol | 1985 | 2025 | after | 40 |
| cinoxacin | 1999 | 0 | - | - |
| cisapride | 2000 | 2000 | same year | 0 |
| clofibrate | 2002 | 1980 | before | 22 |
| danthron | 1987 | 1988 | after | 1 |
| dapiprazole | 2013 | 1995 | before | 18 |
| dexfenfluramine | 1997 | 1998 | after | 1 |
| dextropropoxyphene | 2010 | 1976 | before | 34 |
| diamthazole | 1972 | 1952, 1957 | before | 15-20 |
| dibromsalan | 1975 | 1981 | after | 6 |
| dicumarol | 1955 | 1950 | before | 5 |
| dihydrostreptomycin | 1970 | 1953 | before | 17 |
| dimethylamylamine | 1983 | 2013 | after | 30 |
| diphemanil | 1994 | 1953 | before | 41 |
| diphenidol | 2009 | 1991, 2008 | before | 1-18 |
| dipyrone (metamizole) | 1977 | 2016 | after | 39 |
| dithiazanine iodide | 1964 | 1959 | before | 5 |
| doxylamine | 1983, 2013 | 1983, 2013 | same year | 0 |
| drotrecogin alfa | 2011 | 2003 | before | 8 |
| dydrogesterone | 1993 | 2025 | after | 32 |
| encainide | 1991 | 1990 | before | 1 |
| enoxacin | 1992 | 1988 | before | 4 |
| ***Drug Name (synonym)*** | ***Year of removal*** | ***publication peak*** | ***publication peak: prior or after removal of US market*** | ***difference between removal and publication peak (years)*** |
| etamivan | ? | 1962, 1964, 1965 | ? | ? |
| ethchlorvynol | 1999 | 1964 | before | 35 |
| ethoheptazine | 1984 | 1965 | before | 19 |
| ethyl biscoumacetate | 1999 | 1951 | before | 48 |
| ethyl nitrite (sweet spirits of nitre) | 1980 | 2007 | after | 27 |
| etretinate | 1998 | 1987 | before | 11 |
| fenclozic acid | 1970 | 2013 | after | 43 |
| fenfluramine | 1997, 2024 | 1997 | same year | 0 |
| flosequinan | 1993 | 1993 | same year | 0 |
| gallamine | 2001 | 1971 | before | 30 |
| glafenine | 1992 | 1979 | before | 13 |
| grepafloxacin | 2003 | 2000 | before | 3 |
| hexocyclium | 2006 | 1990 | before | 16 |
| hydrocortamate | 2001 | 1959, 1965, 2025 | unspecific | 24-25 |
| indoprofen | 1984 | 1981 | before | 3 |
| iodamide | 1987 | 1978 | before | 9 |
| iopanoic acid | ? | 1980 | ? | ? |
| iproniazid | 1961 | 1959 | before | 2 |
| levacetylmethadol | 2003 | 1976 | before | 27 |
| levallorphan | 2001 | 1973 | before | 28 |
| levamisole | 2000 | 1979 | before | 21 |
| lomefloxacin | 2009 | 1992 | before | 17 |
| loracarbef | 2006 | 1992 | before | 14 |
| masoprocol | 1996 | 1991 | before | 5 |
| mazindol | 1999 | 1989 | before | 10 |
| mepazine hydrochloride | 1970 | 1958, 1980 | unspecific | 10-12 |
| mephentermine | 2011 | 1963 | before | 48 |
| mesoridazine | 2008 | 1973, 1975 | before | 33-35 |
| metabromsalan | 1975 | 1981, 2019 | after | 6-44 |
| metandienone (methandrostenolone | 1982 | 1966 | before | 16 |
| ***Drug Name (synonym)*** | ***Year of removal*** | ***publication peak*** | ***publication peak: prior or after removal of US market*** | ***difference between removal and publication peak (years)*** |
| methapyrilene | 1979 | 1984 | after | 5 |
| methaqualone | 1982 | 1974 | before | 8 |
| metharbital | 1990 | 1957, 1957, 1981 | before | 9-33 |
| methicillin | 2010 | - | - | - |
| methopholine | 1965 | 1962, 1963, 1967 | before | 2-3 |
| methoxyflurane | 2001, 2005 | 1973 | before | 28-32 |
| metipranolol | 2001 | 1981 | before | 20 |
| metocurine iodide | 2001 | 1985 | before | 16 |
| mibefradil | 1998 | 1998 | same year | 0 |
| mivacurium | 2021 | 1995 | before | 26 |
| moricizine | 2007 | 1986 | before | 21 |
| muzolimine | 1987 | 1985 | before | 2 |
| nalidixic acid | 1992 | 2014 | after | 22 |
| nialamide | 1963 | 1964 | after | 1 |
| nikethamide | 1988 | 1969 | before | 19 |
| nomifensine | 1986, 1992 | 1985 | before | 1-7 |
| novobiocin | 1999, 2011 | 1957 | before | 42-54 |
| oxeladin | 1976 | 2021 | after | 45 |
| oxphenbutazone | 1984 | 1964 | before | 20 |
| oxyphenisatin, oxyphenisation acetate | 1972 | 1980 | after | 8 |
| parecoxib | 2005 | 2006, 2021 | after | 1-15 |
| pargyline | 2007 | 1985 | before | 22 |
| pemoline | 2005 | 1967 | before | 38 |
| pentetrazol (pentylenetetrazol) | 1982 | 2020 | after | 38 |
| pergolide | 2007 | 2002 | before | 5 |
| phenacetin | 1983 | 1964 | before | 19 |
| phenformin | 1978 | 1968 | before | 10 |
| phenolphthalein | 1999 | 1956 | before | 43 |
| phenylpropanolamine | 2000 | 2005 | after | 5 |
| pipamazine | 1969 | 1960 | before | 9 |
| ***Drug Name (synonym)*** | ***Year of removal*** | ***publication peak*** | ***publication peak: prior or after removal of US market*** | ***difference between removal and publication peak (years)*** |
| pipobroman | 2015 | 1998, 2003 | before | 12-17 |
| pirprofen | 1990 | 1985 | before | 5 |
| plicamycin | 2000 | 1972 | before | 28 |
| potassium arsenite | 1980 | 1950, 1985 1988, 2016 | unspecific | 30-36 |
| prenylamine | 1988 | 1968 | before | 20 |
| probucol | 1995 | 1994 | before | 1 |
| quinethazone | 1989 | 1964 | before | 25 |
| rapacuronium | 2001 | 1999, 2001 | prior until same year | 0-2 |
| remoxipride | 1993 | 1990 | before | 3 |
| rimonabant | 2008 | 2008 | same year | 0 |
| ritodrine | 1998 | 1985 | before | 13 |
| rofecoxib | 2004 | 2005 | after | 1 |
| sibutramine | 2010 | 2010 | same year | 0 |
| sitaxentan | 2010 | 2008, 2009 | before | 1-2 |
| sparfloxacin | 2001 | 1999 | before | 2 |
| sparteine | 1979 | 1991 | after | 12 |
| sulfacytine | 2006 | 1976 | before | 30 |
| sulfadimethoxine | 1966 | 2009 | after | 43 |
| sulfamethazine | ? | 2020, 2022 | ? | ? |
| sulfapyridine | 1990 | 2023 | after | 33 |
| sulfathiazole | 1970 | 1946 | before | 24 |
| sulfinpyrazone | 2009 | 1980 | before | 29 |
| suloctidil | 1985 | 1983 | before | 2 |
| suprofen | 1988 | 1983, 1986 | before | 2-5 |
| tacrine | 2013 | 1996 | before | 17 |
| tegaserod | 2007, 2019 | 2007 | same year | 0 |
| telaprevir | 2015 | 2014 | before | 1 |
| temafloxacin | 1992, 1997 | 1991 | before | 1-6 |
| terfenadine | 1998 | 1993 | before | 5 |
| thenalidine | 1963 | 1984 | after | 21 |
| thiabendazole | 1995 | 2021, 2025 | after | 26-30 |
| thiethylperazine | 2009 | 1963 | before | 46 |
| thiopental | 2011 | 1993 | before | 18 |
| ***Drug Name (synonym)*** | ***Year of removal*** | ***publication peak*** | ***publication peak: prior or after removal of US market*** | ***difference between removal and publication peak (years)*** |
| ticrynafen  (tienilic acid) | 1980, 1996 | 1979 | before | 42736 |
| tocainide | 2003 | 1985 | before | 18 |
| tolazoline | 2002 | 1981 | before | 21 |
| tribromsalan | 1975 | 1981 | after | 6 |
| tridihexethyl | 2012 | 1964, 1965 | before | 48-49 |
| trilostane | 1994 | 2011 | after | 17 |
| trioxsalen | 2002 | 1982 | before | 20 |
| triparanol | 1962 | 1964 | after | 2 |
| troglitazone | 2000 | 2000 | same year | 0 |
| trovafloxacin | 2006 | 2000 | before | 6 |
| tryptophan | 1989 | - | - | - |
| tyloxapol | 2003 | 1995 | before | 8 |
| valdecoxib | 2005 | 2005 | same year | 0 |
| vidarabine | 2006 | 2011 | after | 5 |
| zalcitabine | 2006 | 2007 | after | 1 |
| zimelidine | 1983 | 1983 | same year | 0 |
| zomepirac | 1983 | 1983 | same year | 0 |
